# Supplementary figures and images for: Environmental DNA: A promising factor for tuberculosis risk assessment in multi-host settings
Source: PLoS One. 2020 May 29;15(5):e0233837. doi: 10.1371/journal.pone.0233837 (PMC7259669; doi:10.1371/journal.pone.0233837)

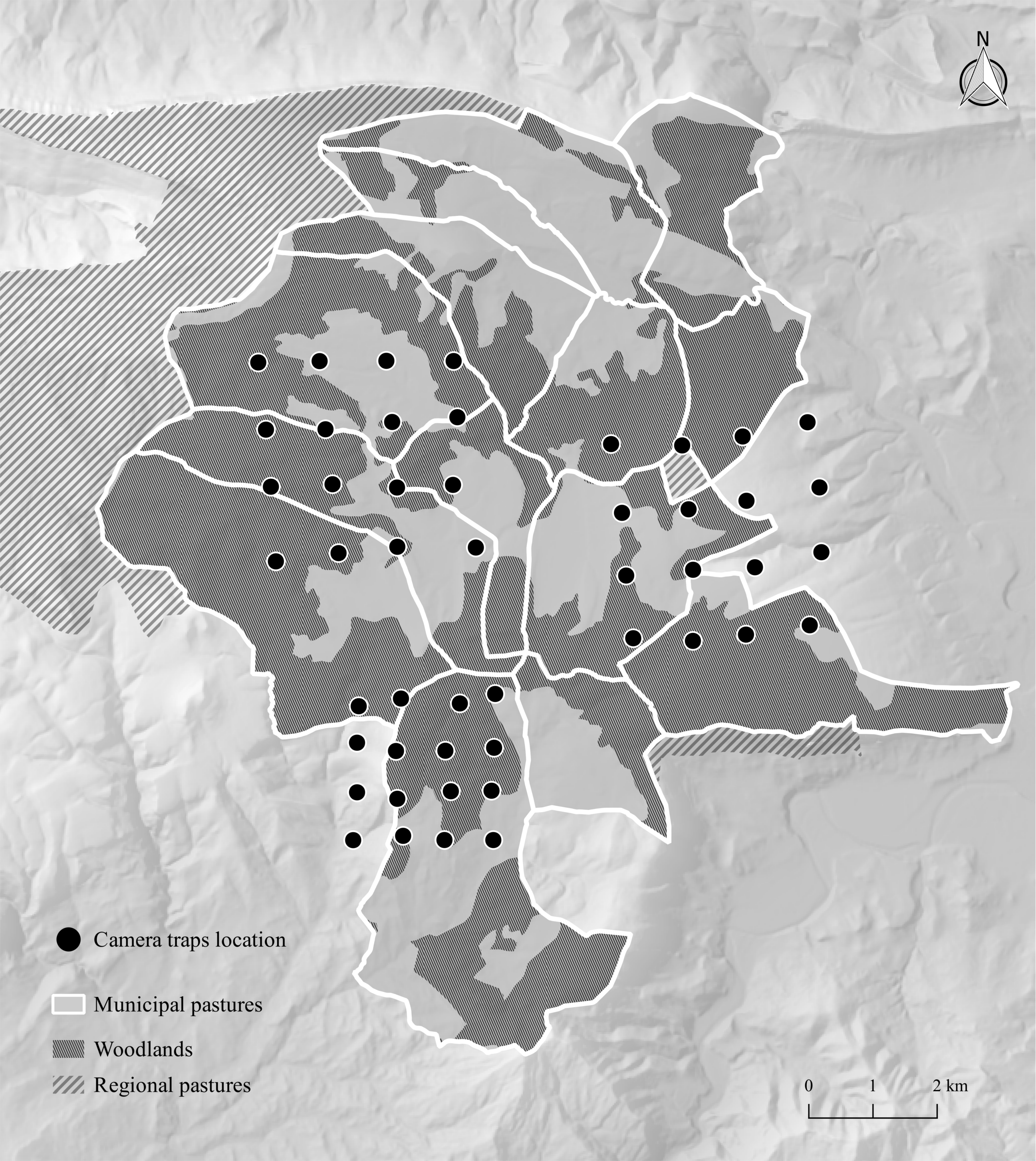

Supplement: S1 Fig — (TIF) [file pone.0233837.s001.tif]
