# Supplementary material for: Environmental DNA: A promising factor for tuberculosis risk assessment in multi-host settings
Source: PLoS One. 2020 May 29;15(5):e0233837. doi: 10.1371/journal.pone.0233837 (PMC7259669; doi:10.1371/journal.pone.0233837)
Supplement: S1 Table — Each column shows the positive animals (cattle) in the respective years, categorized by diagnostic technique according to the following color code: Positive Tuberculin Intradermal Reaction (red); Positive Interferon-γ Gamma Release Assay (blue); Positive Slaughter (Lesions) (yellow); Positive Culture (green). If the same animal tested positive for more than one diagnostic test, only the color of the most restrictive technique is shown. The last column shows the environmental MTC DNA sampling results, categorized as MTC (Mycobacterium tuberculosis Complex), MAC (Mycobacterium avium Complex), Mycobacterium sp. (genus Mycobacterium not belonging to MTC or MAC) and 0 (negative or not conclusive). (PDF) [file pone.0233837.s002.pdf]

FARM ID	TB 2001	TB 2002	TB 2003	TB 2004	TB 2005	TB 2006	TB 2007	TB 2008	TB 2009	TB 2010	TB 2011	TB 2012	TB 2013	TB 2014	TB 2015	TB 2016	Environmental MTC DNA
ID1	0	0	0	4	0	0	0	3	0	0	0	0	2	0	1	1	MTC/MTC
ID2	0	0	0	0	0	0	0	0	0	0	0	1	1	0	0	0	0
ID3	0	0	0	0	0	0	0	0	0	2	1 + 6	0	0	0	0	3	0
ID4	0	0	0	0	0	0	0	0	0	0	0	0	0	0	0	0	0
ID5	0	0	0	0	0	0	0	0	0	0	0	0	0	0	0	0	MTC
ID6	0	0	0	2	0	0	0	0	0	0	0	0	1 + 3	11	0	1	0
ID7	0	0	0	0	0	0	0	0	0	0	0	0	0	0	0	0	0
ID8	0	0	0	0	0	0	0	0	0	0	0	0	1	0	0	4	MTC/MAC
ID9	0	0	4	0	0	0	0	0	0	4	3	0	0	1	2 + 2	0	0
ID10	0	0	1	0	0	0	0	0	0	0	2	0	0	0	0	2	MTC/*Mycobacterium sp.*
ID11	NA	NA	NA	0	0	0	0	0	0	0	0	0	1 + 1	13	0	0	0
ID12	NA	NA	NA	0	0	0	0	0	0	1	0	0	0	2	1 + 1	0	0
ID13	NA	NA	NA	0	0	0	1	0	0	0	0	1	0	0	0	0	MTC
ID14	0	0	5	2	0	0	0	0	0	6	6	0	0	0	0	0	MTC/*Mycobacterium sp.*
ID15	NA	NA	0	0	11	0	5	0	0	0	0	0	0	8 + 2	11 + 1	1 + 9	0
ID16	NA	NA	0	0	0	0	0	0	0	1	0	0	0	0	0	3	0
ID17	NA	NA	NA	0	0	0	0	0	0	0	0	0	0	0	0	0	MTC
ID18	NA	NA	0	0	0	0	0	0	0	0	0	0	0	0	0	0	MTC
ID19	0	0	0	0	0	0	0	0	0	0	0	0	0	0	1	0	MTC
ID20	NA	NA	NA	0	0	0	0	0	0	0	0	0	0	0	1	0	MTC
ID21	NA	NA	NA	0	0	0	0	0	0	1	0	0	0	0	0	0	0
ID22	NA	NA	NA	0	0	0	0	0	0	0	1	5 + 1	0	0	0	4 + 6	MTC/*Mycobacterium sp.*
ID23	0	0	0	0	0	0	0	0	0	5	3 + 2	0	0	0	0	0	MTC
ID24	NA	NA	1	0	0	0	0	0	0	0	0	0	1	1 + 19	4	1 + 7	0

Positive Tuberculin Intradermal Reaction; Positive Interferon-γ Gamma Release Assay; Positive Slaughter (Lesions); Positive Culture.

MTC (*Mycobacterium tuberculosis* Complex); MAC (*Mycobacterium avium* Complex); *Mycobacterium sp.* (genus *Mycobacterium* not belonging to MTC or MAC).

If the same animal has tested positive for more than one diagnostic test, only the color of the most restrictive technique is shown.
