# Supplementary material for: Environmental DNA: A promising factor for tuberculosis risk assessment in multi-host settings
Source: PLoS One. 2020 May 29;15(5):e0233837. doi: 10.1371/journal.pone.0233837 (PMC7259669; doi:10.1371/journal.pone.0233837)
Supplement: Attachment — Submitted filename: Response to reviewers.pdf [file pone.0233837.s003.pdf]

PONE-D-19-20068

Environmental DNA points at shared pastures and proximity to woodlands as tuberculosis risk factors for cattle in multi-host settings

PLOS ONE

Journal Requirements:

1. When submitting your revision, we need you to address these additional requirements.

Please ensure that your manuscript meets PLOS ONE's style requirements, including those for file naming. The PLOS ONE style templates can be found at http://www.journals.plos.org/plosone/s/file?id=wjVg/PLOSOne_formatting_sample_main_body.pdf and http://www.journals.plos.org/plosone/s/file?id=ba62/PLOSOne_formatting_sample_title_authors_affiliations.pdf

- ➔ OK, done.

2. Thank you for stating the following in the Competing Interests section:

"I have read the journal's policy and the authors of this manuscript have the following competing interests: MB and SABIOTEC are consultants and offer risk assessment at the wildlife-livestock interface."

Please confirm that this does not alter your adherence to all PLOS ONE policies on sharing data and materials, by including the following statement: "This does not alter our adherence to PLOS ONE policies on sharing data and materials." (as detailed online in our guide for authors http://journals.plos.org/plosone/s/competing-interests). If there are restrictions on sharing of data and/or materials, please state these. Please note that we cannot proceed with consideration of your article until this information has been declared.

Please include your updated Competing Interests statement in your cover letter; we will change the online submission form on your behalf.

- ➔ OK, done. The following state is included in the cover letter: "This does not alter our adherence to PLOS ONE policies on sharing data and materials."

3. We note that Figure(s) 1 in your submission contain [map/satellite] images which may be copyrighted. All PLOS content is published under the Creative Commons Attribution License (CC BY 4.0), which means that the manuscript, images, and Supporting Information files will be freely available online, and any third party is permitted to access, download, copy, distribute, and use these materials in any way, even commercially, with proper attribution. For these reasons, we cannot publish previously copyrighted maps or satellite images created using proprietary data, such as Google software (Google Maps, Street View, and Earth). For more information, see our

copyright guidelines: http://journals.plos.org/plosone/s/licenses-and-copyright.

We require you to either (1) present written permission from the copyright holder to publish these figures specifically under the CC BY 4.0 license, or (2) remove the figures from your submission:

a) You may seek permission from the original copyright holder of Figure(s) [#] to publish the content specifically under the CC BY 4.0 license.

We recommend that you contact the original copyright holder with the Content Permission Form (http://journals.plos.org/plosone/s/file?id=7c09/content-permission-form.pdf) and the following text:
"I request permission for the open-access journal PLOS ONE to publish XXX under the Creative Commons Attribution License (CCAL) CC BY 4.0 (http://creativecommons.org/licenses/by/4.0/). Please be aware that this license allows unrestricted use and distribution, even commercially, by third parties. Please reply and provide explicit written permission to publish XXX under a CC BY license and complete the attached form."

Please upload the completed Content Permission Form or other proof of granted permissions as an "Other" file with your submission.

In the figure caption of the copyrighted figure, please include the following text: "Reprinted from [ref] under a CC BY license, with permission from [name of publisher], original copyright [original copyright year]."

b) If you are unable to obtain permission from the original copyright holder to publish these figures under the CC BY 4.0 license or if the copyright holder's requirements are incompatible with the CC BY 4.0 license, please either i) remove the figure or ii) supply a replacement figure that complies with the CC BY 4.0 license. Please check copyright information on all replacement figures and update the figure caption with source information. If applicable, please specify in the figure caption text when a figure is similar but not identical to the original image and is therefore for illustrative purposes only.
The following resources for replacing copyrighted map figures may be helpful:

USGS National Map Viewer (public domain): http://viewer.nationalmap.gov/viewer/
The Gateway to Astronaut Photography of Earth (public domain): http://eol.jsc.nasa.gov/sseop/clickmap/
Maps at the CIA (public domain): https://www.cia.gov/library/publications/the-world-factbook/index.html and https://www.cia.gov/library/publications/cia-maps-publications/index.html
NASA Earth Observatory (public domain): http://earthobservatory.nasa.gov/
Landsat: http://landsat.visibleearth.nasa.gov/
USGS EROS (Earth Resources Observatory and Science (EROS) Center) (public domain): http://eros.usgs.gov/#
Natural Earth (public domain): http://www.naturalearthdata.com/

➔ OK. All material used to build the map contained in "Figure 1" is public and freely available online. Specifically, the altitude raster map used to build the

background is available on the IGN web page (https://www.ign.es/web/cbg-area-cartografia) from the "Ministerio de Fomento, Gobierno de España", and the administrative shapefiles used to delimit the study area are available on the DGC web page (http://www.sedecatastro.gob.es/) from the "Ministerio de Hacienda, Gobierno de España". The map itself was built using QGIS Desktop 2.14 software, a free and Open Source Geographic information System (https://qgis.org/en/site/).

Comments to the Author

1. Is the manuscript technically sound, and do the data support the conclusions?

The manuscript must describe a technically sound piece of scientific research with data that supports the conclusions. Experiments must have been conducted rigorously, with appropriate controls, replication, and sample sizes. The conclusions must be drawn appropriately based on the data presented.

Reviewer #1: Yes

Reviewer #2: Partly

---

2. Has the statistical analysis been performed appropriately and rigorously?

Reviewer #1: I Don't Know

Reviewer #2: No

---

3. Have the authors made all data underlying the findings in their manuscript fully available?

The PLOS Data policy requires authors to make all data underlying the findings described in their manuscript fully available without restriction, with rare exception (please refer to the Data Availability Statement in the manuscript PDF file). The data should be provided as part of the manuscript or its supporting information, or deposited to a public repository. For example, in addition to summary statistics, the data points behind means, medians and variance measures should be available. If there are restrictions on publicly sharing data—e.g. participant privacy or use of data from a third party—those must be specified.

Reviewer #1: Yes

Reviewer #2: Yes

---

4. Is the manuscript presented in an intelligible fashion and written in standard English?

PLOS ONE does not copyedit accepted manuscripts, so the language in submitted articles must be clear, correct, and unambiguous. Any typographical or grammatical errors should be corrected at revision, so please note any specific errors here.

Reviewer #1: Yes

Reviewer #2: Yes

---

5. Review Comments to the Author

Please use the space provided to explain your answers to the questions above. You may also include additional comments for the author, including concerns about dual publication, research ethics, or publication ethics. (Please upload your review as an attachment if it exceeds 20,000 characters)

Reviewer #1: Here are some comments about your work that I would like you to consider:
Line number (LN) 3: The authors must write accent mark in: Martínez-Guijosa

- ➔ OK, done.

In the abstract: The authors must remove the abbreviations: TB (tuberculosis) and ED (epidemiological diagnosis)

- ➔ OK, done.

LN42: The authors must write: Mycobacterium instead of M.

- ➔ OK, done.

LN80: The authors must write: Materials (in plural)

- ➔ OK, done.

LN103: The authors must change: …described by Rowcliffe et al. 2008 [21], for: …described previously [21]

- ➔ OK, done.

LN100-110: The authors must indicate the study period (2012-2016 also or only 2015/2016? Not specified)

- ➔ OK, done. In lines 102-103 is stated "installed from September 5 to October 12 (2016)" to specify the camera trapping study period. In line 131 is stated "All

the work performed on the farms was carried out between September 2016 and January 2017" to specify on-farm study period.

LN110: The authors must write: and submitted for bacteriological culture and molecular identification in a...

- ➔ OK, done.

LN115: The authors must clarify how many cattle (since if there were a total of 3,100 cattle in 36 herds, how many cattle were in the 24 herds were studied?) and goats (a total of 309 goats?) are tested for TB.

- ➔ Thank you for the comment. In lines 118-119 is stated: "The 24 herds selected for the study added 2,786 cattle (89.87% of the total cattle in the study area)", to specify how many cattle were in the 24 selected herds.

  Yes, a total of 309 goats were tested for TB in the year 2016, since "All cattle and goats are tested for TB on an annual basis by means of the official single intradermal tuberculin test according to the Spanish bovine tuberculosis eradication program…".

LN116 and LN117: The authors must write: Spanish Eradication Program of Bovine Tuberculosis just like they have written in LN127.

- ➔ OK, done.

LN117: The authors must clarify if in Spanish Eradication Program of Bovine Tuberculosis (year 2018) were available the data for cattle and goats for the period 2012-2016 or just the cattle's data.

- ➔ OK, done. Thank you for the comment. The Spanish Eradication Program of Bovine Tuberculosis explains the protocols and procedures followed in Spain to achieve the eradication of Bovine Tuberculosis in cattle and goats with contact with cattle (directly or through common pastures) or those flocks considered as a possible source of infection to cattle. Information about the prevalence of the disease in cattle herds at national, provincial and regional levels are given in this annual report, including the period of this study (2012-2016). Since prevalence and incidence of TB in goats is not included in this Spanish program, the Government of Navarra, which actively participated in the conduct of this study, provided them.

  In line 121 is stated: "…and data for the period 2012-2016 was, therefore, kindly provided by the Government of Navarra" to clarify this fact.

In the Table 1: The authors should analyse if the expression "Freq. sees roe deer" refers to seeing roe deer (*Capreolus capreolus*) or actually they mean red deer (Cervus elaphus). If it is not a spelling mistake, the authors must make reference in the introduction (LN49-LN54) to the relationship between roe deer and tuberculosis, e. g. "the roe deer (*Capreolus capreolus*) are not a significant TB reservoir" (Balseiro A,

Oleaga Á, Orusa R, Robetto S, Domenis L, Zoppi S, et al. Tuberculosis in roe deer from Spain and Italy. Vet Rec. 2009;164: 468-470). "However, once infected, it develops lesions reflecting a clear ability for bacterial excretion and therefore transmission to other species, most likely by indirect contact. It could thus be a spillover host included in a multi-host component reservoir in endemic areas. Consequently, passive surveillance is essential to detect infection and to implement specific management to limit interactions with cattle, where infected roe deer are found" (Lambert S, Hars J, Réveillaud E, Moyen JL, Gares H, Rambaud T, et al. Host status of wild roe deer in bovine tuberculosis endemic areas. Eur J Wildl Res. 2017;63: 15).

- ➔ OK, done. Thank you for the comment and the useful recommendations. It is not a spelling mistake, it refers to *Capreolus capreolus* sights, since *Cervus elaphus* in completely absent in the study area.

  In lines 49-54, the reference to wild TB reservoirs in Spain is only briefly explained at geographical level. However reference [7] (Gortázar C, Fernández-Calle LM, Collazos-Martínez JA, Mínguez-González O, Acevedo P. Animal tuberculosis maintenance at low abundance of suitable wildlife reservoir hosts: A case study in northern Spain. Prev Vet Med. 2017;146: 150–157) include roe deer in its sampling efforts.

  In lines 55-66 the role of wild reservoirs in the Atlantic Spain was explained at species level, so the following sentence is now added in lines 63-65 to clarify the potential role of roe deer in TB transmission: "Roe deer (*Capreolus capreolus*), is an abundant cervid in northern Spain [14], and despite cases of infected roe deer have been evidenced, it does not appear to be a significant reservoir of TB [15,16]". Suggested references included.

LN142 and LN143: The authors must clarify if on each farm, each of the two sponges were used on the back of a single animal (1 sponge / 1 animal) or the same sponge was scrubbed ten times on the back of the two randomly chosen cattle (1 sponge / 2 animals). In addition, the authors must explain why they chose the back and not another anatomical region of the animal such as the head or legs to take the samples of environmental DNA.

- ➔ Thank you for the comment. Two sponges were used per farm and in two different animals. Therefore, two animals provide information about the environmental DNA sampling. It has been included in line146-147 "On each farm, two sponges were scrubbed ten times on the scapula regions of two randomly chosen cattle (one sponge by animal). The scapula region, and no other anatomical region of the animal, was chosen because it is the most accessible area during cattle handling which is relatively close to the brisket, one of the preferred areas for sampling pathogens on cattle hides [28]. In addition, it is a large and quasi-horizontal area, where dust particles can easily deposit."

LN149: The authors must write the primers and probe used to detect IS6110 or add another reference along with the [25].

- ➔ Ok, done. Information about the sequences of primers and probe has been included in the manuscript in lines 155-157 as follows: "A real time PCR with which to detect IS*6110* was used to detect MTC in the samples, using the primers (forward 5'- GGTAGCAGACCTCACCTATGTGT-3'; reverse 5'-AGGCGTCGGTGACAAAGG-3') and probe (5' FAM-CACGTAGGCGAACCC-MGB-NFQ 3') described previously [30].

LN166: The authors must write: The local host community is composed of XXXX cattle, just like they have written in LN115.

- ➔ OK, done.

LN180-LN182: The authors must indicate how many MTC isolates were obtained in the study in each animal species, e. g. "All cattle (N= XXX), sheep (N= 4), wild boar (N= 5) and badger (N= 1) isolates obtained in the study were identified as M. bovis and were classified with SB0134 spoligotype (according to the Mbovis.org database)".

- ➔ OK, done.

LN187-190: Authors should refer to the results observed in: "A conventional PCR was also used to discard the presence of Mycobacterium avium subspecies [26]".

- ➔ Thank you for this comment. Only one positive results to Mycobacterium avium complex was detected. It was included in the manuscript in lines 198-199: "*Mycobacterium avium* subspecies was only detected in one sample from a farm where the additional sponge was only positive to MTC".

LN216 and LN217. The authors must explain where they obtained these data ("also about 23 to 43 MTC infected wild boar -at a minimum prevalence of 10%-") because previously they indicated: "With regard to wildlife, we sampled 57 hunter-harvested wild boar. The TB-compatible lesion prevalence was 54% (31/57) and the infection prevalence confirmed by culture was 8.8% (5/57)".

- ➔ Thank you for the comment. 10% was selected as an approximate minimum TB prevalence in the study area to exemplify the potential numbers of infected wild boars in that area, because of simplicity. Nevertheless, is true that, to be more accurate, we could use the exact percentage presented in our results. The numbers have been recalculated with this 8.8% to solve this problem (lines 224-225: "…20 to 38 MTC infected wild boar …").

LN251-LN257: The authors must explain why the environmental DNA samples that proved to be positive for the presence of MTC were not analyzed by spoligotyping as did Barbier el at. 2016 [26], especially considering that "all cattle, sheep, wild boar and badger isolates obtained in the study were identified as M. bovis and were classified with SB0134 spoligotype".

➔ Thank you for your comments. Barbier et al. used the 6 positive samples of their study. They could only provide a complete profile in one of the samples, 3 profiles were not completed and the 2 samples left were negative. They did not provide information about the Ct values of the samples at the real time PCR used. In our hands, the Ct values obtained for most of the samples were > 35 and it cannot allow the minimum quantity and quality of DNA for spoligotyping purposes.

LN272-LN274: The authors must write: Gortázar C, Che Amat A, O'Brien DJ. Open questions and recent advances in the control of a multi-host infectious disease: Animal tuberculosis. Mamm Rev. 2015;45: 160–175. doi:10.1111/mam.12042

➔ OK, done.

LN277: Authors should change the URL and put: https://www.mapa.gob.es/es/ganaderia/temas/sanidad-animal-higiene-ganadera/pnetb_2018_tcm30-436761.pdf

➔ OK, done.

LN278: The authors must write accent mark in: Liébana, Gómez-Mampaso, Galán

➔ OK, done.

LN 283: The authors must write: infections (no italic letter)

➔ OK, done.

LN 296: The authors must delete: Khudyakov YE, editor.

➔ OK, done.

LN297: The authors must delete: –e71074

➔ OK, done.

LN302 and LN303: The authors must delete: Fooks A, editor. – e18058

➔ OK, done.

LN318 and LN319: Authors should change the URL and put: https://www.mapa.gob.es/es/ganaderia/temas/sanidad-animal-higiene-ganadera/patubes2017_3_tcm30-378321.pdf

➔ OK, done.

LN325 and LN326: The authors must write: Wildlife/Livestock Interface. Transbound Emerg Dis. 2017;64: 1148–1158. doi:10.1111/tbed.12480

➔ OK, done.

LN331: The authors must write: Mycobacterium bovis (with italic letter)

➔ OK, done.

LN347: The authors must delete: Inacio J, editor. –e88824

➔ OK, done.

LN373: The authors must write accent mark in: Pérez A,

➔ OK, done.

Reviewer #2: This is an interesting paper describing the relationship between on-animal environmental MTC DNA detection and bovine TB risk in a multi-host scenario. Detection of environmental MTC DNA in cattle herds is an interesting tool, however I think results obtained in this paper does not completely support conclusions and some important issues need to be considered for publication
The main concerns about this paper are:
- Interpretation of results: Authors conclude that on-animal MTC DNA detection could be a good proxy for TB risk in cattle herds and that it could be used as an epidemiological tool to understand unexpected results.
To support this conclusion it could be expected an association between the detection of MTC DNA and the presence of new TB-positive animal at heard level. However, this hypothesis has not been assessed in this work. Furthermore, there is not association between herds where MTC DNA was detected and herd with higher risk of TB. MTC-DNA detection in cattle herds was related to the use of communal pasture, altitude and distance to woodland, but it does not mean that this technique was related with the risk of TB infection. Other risk factors also related with TB risk like the abundance of wild boar or the presence of risk points, seem not to be associated with MTC-DNA detection, and it should be discussed.

➔ Thank you for the comment, we share your concern. In this study, what we propose is that this tool can be used as an indicator of the risk of environmental MTC DNA exposure, and by extension, it has the potential to indicate the risk of exposure to TB-causative agents, since these belong to the MTC. However, the possible interference with non-TB-causing MTC members is possible, and therefore we have maintained prudence in our discussion, referring at all times to the risk of exposure to MTC DNA, as well as the circumstantial utility of this tool, which must still be tested in different scenarios to determine its true potential to help in epidemiological diagnoses and to solve sanitary problems at the landscape or herd level. Even so, we firmly rely on the potential of this tool, since, in this specific case, it has helped to identify certain risk factors in which authorities and farmers can work and stay alert. Well informed about the scope of our results and their limitations, the stakeholders involved in the problem have information with a scientific basis to try to solve their problems, which,

with the information they had so far, could not solve despite their professionalism and their strictness.
On the other hand, when we state that this tool can help to understand unexpected results, we do not mean that it can help the individual diagnosis of animals, but, as is the case, can help to interpret results that, only with Official data are hardly interpretable, and therefore, they give no clues to develop specific actions to solve the problem.
In this sense, the lack of association between the detection of MTC DNA and the presence of new TB-positive animal at herd level can be due to various factors. First, it may be conditioned by the sensitivity problems of the diagnostic tests used (de la Rua-Domenech et al., 2006), especially taking into account the low incidence of TB in the area, and, second, it may be due to limitations (previously discussed) of the environmental DNA detection tool used in this study. However, given the impossibility of unravelling the reason for this issue, we believe that this lack of association shows how the use of new epidemiological tools that provide complementary data to existing epidemiological information can help solve specific problems in complex epidemiological scenarios.

Regarding the “abundance” of wild boar, in the first place we do not know exactly the role that this species plays in this environment, so it is possible that its potential as a reservoir and transmitter of the disease is associated with specific areas (such as the regional pastures of the Sierra de Urbasa-Andía, where hunting is not allowed), or the access of these animals to specific areas of the facilities during the winter, thus confounding their effect with the significant variables related with the use of regional pastures or the distance of the facilities to the forest, respectively.
Secondly, it is important to remark that it is not “abundance” of wild boar, but the perception of each farmer of the presence of the species. This measure is individual and subjective, and, despite useful in some specific cases, these particular discrepancies can invalidate the potential effect of this variable in this particular case.

As for the risk points, the number of potential interaction risk points does not have to imply a greater risk of exposure to livestock, since, depending on the nature of the point and the use made by the animals of the different species potentially involved in the epidemiological system, accessibility to a single risky point may pose a greater risk than access to several of them.

- Specificity of the technique: In discussion section the authors assume that the PCR technique used in this work could have small risk of cross reaction with some environmental mycobacteria or with other agents. But, how small is this risk in an scenario where environmental mycobacteria have been isolated repeatedly isolated from cattle and wildlife? Could the specificity be improved using confirmative PCR assays based on exclusive M. bovis gene?
Furthermore, about the sensitivity, could the absence of MTC DNA be ensured testing only two animals per herd?

➔ Thank you for the comment.
    1. Up to know, we cannot quantify the risk of cross reaction with some environmental mycobacteria.

2. The idea to use an additional real time PCR to confirm the previous result was evaluated. However, in most cases, the Ct values of the IS6110 PCR was high (Ct>35) and, taking into account that some *M. bovis* has several copies of the IS element, the sensitivity of another real time PCR could be lower than the IS6110. However, we did not include in this study the confirmatory PCR so no data are available.
3. No. Since 24 farms were studied, due to logistic reasons, and with the scientific objective of testing this new methodology to detect mycobacteria in the field, we decided to include only two sponges per farm, and see the results. We cannot assure that the absence of detection of environmental DNA in two samples represents the situation in the farm, but based on the results, it is a proxy or estimation of the farm MTC exposure risk.

- Cost-benefits of on-animal MTC detection: Authors should discuss whether this technique is appropriate regarding to cost-benefits criteria.

➔ Thank you for the comment. This methodology could be used in different scenarios, but it could be useful, for example, when: 1) recurrent TB is present in a farm or 2) after stamping out and disinfection of the farm to avoid reinfection of animals from environmental sources. Cost-benefit balance when using this technique will depend on the particular situation of the farm, and the balance will be positive in cases where the persistence of mycobacteria represents a problem for the economic and/or sanitary viability of the farm. However, it is not possible to establish a confident calculation of the generalized or commercial implementation of this technique, since now, the actual potential of its use in various scenarios where it could also be useful is unknown.
To discuss this issue, we added the following paragraph in lines 259-263: "Additional research is needed to verify the usefulness of this novel technique in different situations where its implementation has the potential to support the economic and sanitary viability of livestock farms, such as, for example, in farms with recurrent TB cases, or after stamping out and disinfection of the farm in order avoid animal reinfection from environmental sources."

Other issues about the paper are:
- Lines 178-179: Although the prevalence of tb-like lesions in wild boar was a 54%, the infection only was confirmed in a 8.8%. I think this issue should be discussed as an unexpected result. Could infections with other agents related with MTC produce this kind of lesion?

➔ Thank you for the comment. Yes, as explained in Bollo et al. 2000, this kind of lesions can be produced by other mycobacteria (i.e. *M. avium* complex), or by other pathogens, such as *Rhodococcus equi*. Additionally, it was previously

reported that the use of a single diagnosis *post-mortem* test on hunter-harvested wild boar, can underestimate the true prevalence, and that despite bacteriological culture is the reference test for TB diagnosis, it can generate false-negative results, and this should be considered when interpreting data (Santos et al. 2010). Nevertheless, we were aware of the possible biases in the prevalence obtained by any method, and therefore we considered the most conservative value as the reference value for later discussion, that is, the one based on culture. Regarding the discussion of the nature of these results, it is not the objective of this study, since the prevalence in wild boar is provided as descriptive data to provide the reader with general information on the epidemiological context of TB in this area.

- Lines 184-486: Which scientific criteria have been used to stablish hard or low TB risk herds? Perhaps, low TB risk herds could be those without positive animals. Furthermore, in order to avoid theoretical classification, quantitative variable would be used to measure TB risk (e.g. number of positive animals or mean prevalence in the last 10 years, etc.)

➔ Thank you for the comment. As stated in lines 193-194: "…the farms were classified as farms with a higher risk, those that were positive to TB for at least two years (consecutive or otherwise) (n=10), and farms with a lower risk, i.e., those with reactors in one year or without reactors (n = 14), in the period of study (2012-2016)."
Since incidence and prevalence was generally very low in all herds (actually Navarra is a low prevalence region compared to other Spanish regions), and the temporal pattern of herd prevalence was apparently random, we decided to establish a criterion based on the positivity at herd level within the last five years (based on the "Historical persistence" described by Martínez-López et al. 2014 as one of the main factors for TB occurrence and persistence), rather than focusing on the number of positive animals in the herd.

- Lines 188-190: When were samplings to detect MTC environmental DNA carried out (simultaneously, in different years…). Results of MTC samplings should be added to s1 table.

➔ Thank you for the comment. The following state has been added in line 144 in order to clarify this point: "During January 2017, samples of environmental DNA were taken from cattle from 24 farms."

Results of MTC samplings now added to S1 table and S1 table caption has been modified.

- Lines 193-194: Do cattle herds sharing the same municipal pasture really coincide as regards their MTC DNA detection status? I think it should be clarify. In Figure 1 I count

five municipalities with more than one cattle herds and only in three of them I can see a complete coincidence in MTC status.

- ➔ Thank you for the comment. No, Fisher's test not reveal significant association between municipal pasture groups and MTC DNA detection status (P = 0.2346). To avoid confusions, the sentence "However, Fig 1 suggests that herds of cattle sharing the same municipal pastures often coincide as regards their MTC DNA detection status" has been removed.

- Lines 195-196. Are there any correlation between altitude and woodland distance? I could be an interesting point to assess.

- ➔ Thank you for the comment. Yes, there is correlation between altitude and woodland distance (Pearson's *r* = 0.45; P= 0.02402).
  The following sentence has been modified in lines 243-244 to clarify this question: "In addition, farm altitude and distance to woodland proved significant (both factors are correlated with each other; Pearson's r = 0.45; P= 0.02402). We suggest that closeness to woodland (and in this case, altitude) is a proxy for the risk of contact with wildlife MTC hosts such as badgers and wild boar."

- Line 233. Was the use of communal pastures really identified as a risk factor for TB in cattle in this work?

- ➔ Thank you for the comment. No, only one kind of communal pastures (regional pasture) was identified as a risk factor. In lines 234-240, the word "communal" was used erroneously in reference to the specific regional pasture in the study area. It has been addressed in lines 234, 237, 238 and 240. The use of "communal" in lines 241 and 242 is correct, since it refers to communal pastures in a general sense, as pastures shared by several farmers.

---

6. PLOS authors have the option to publish the peer review history of their article (what does this mean?). If published, this will include your full peer review and any attached files.

If you choose "no", your identity will remain anonymous but your review may still be made public.

Do you want your identity to be public for this peer review? For information about this choice, including consent withdrawal, please see our Privacy Policy.

Reviewer #1: Yes: José Manuel Benítez-Medina

Reviewer #2: No
