# Supplementary material for: Environmental DNA: A promising factor for tuberculosis risk assessment in multi-host settings
Source: PLoS One. 2020 May 29;15(5):e0233837. doi: 10.1371/journal.pone.0233837 (PMC7259669; doi:10.1371/journal.pone.0233837)
Supplement: Attachment — Submitted filename: PONE-D-19-20068_R2_Rev.pdf [file pone.0233837.s005.pdf]

PLOS ONE

Environmental DNA points at shared pastures and proximity to woodlands as tuberculosis risk factors for cattle in multi-host settings

--Manuscript Draft--

Manuscript Number:	PONE-D-19-20068R2
Article Type:	Research Article
Full Title:	Environmental DNA points at shared pastures and proximity to woodlands as tuberculosis risk factors for cattle in multi-host settings
Short Title:	Environmental DNA as epidemiological tool
Corresponding Author:	Jordi Martinez-Guijosa<br>Instituto de Investigacion en Recursos Cinegeticos<br>Ciudad Real, Castilla-La Mancha SPAIN
Keywords:	environmental DNA; Epidemiological diagnosis; Multi-host infection; Non-invasive sampling; On-farm risk assessment; Wildlife-livestock interface; Mycobacterium tuberculosis complex
Abstract:	Attaining and maintaining the Official Tuberculosis Free status continues to be a challenge when several domestic and wild hosts contribute to the maintenance of the Mycobacterium tuberculosis complex (MTC). Local tuberculosis hotspots are sometimes identified in cattle in low-prevalence regions. We have, therefore, studied one such a hotspot in depth in order to produce an epidemiological diagnosis. Host population size and MTC prevalence were estimated in selected wildlife and in livestock, while on-cattle environmental DNA detection was additionally used as a proxy for exposure risk at the farm (herd) level. Positive skin test reactors were found on 16 of the 24 cattle farms studied in the period 2012-2016. Although all goats tested negative to the skin test during this period, MTC was confirmed in four sheep at slaughter, thus indicating an unknown infection prevalence in this host species. With regard to wildlife, wild boar (Sus scrofa) MTC infection prevalence based on culture was 8.8%, and the only road-killed badger (Meles meles) submitted for culture tested positive. Two criteria were employed to divide the cattle farms into higher or lower risk: tuberculosis testing results and environmental DNA detection. Environmental MTC DNA detection yielded significant differences regarding “use of regional pastures” and “proximity to woodland”. This study suggests that on-animal environmental DNA sampling can help in assessing contact risk with MTC in livestock, at the herd level. This tool opens up new avenues for epidemiological research in complex, multi-host settings.
Order of Authors:	Jordi Martínez-Guijosa
	Beatriz Romero
	José Antonio Infantes-Lorenzo
	Elena Díez
	Mariana Boadella
	Ana Balseiro
	Miguel Veiga
	David Navarro
	Inmaculada Moreno
	Javier Ferreres
	Mercedes Domínguez
	Cesar Fernández
	Lucas Domínguez
	Christian Gortázar

Powered by Editorial Manager® and ProduXion Manager® from Aries Systems Corporation

Opposed Reviewers:	
Response to Reviewers:	Reviewer #1: Thanks for including the suggestions. I believe that paper is now better understood. In any case, as you have clarified, this research is especially a methodological work that must be continued to clarify some issues and prove their true usefulness. Please review references 1 (2015; 45: 160–175.) and 3 (name bacteria in italics).<br>->We appreciate the review. Changes made.<br><br>Reviewer #2: Dear authors,<br>Many thanks for responding all my comments included in the first round. I maintain that environmental MTC DNA detection is a promising and an interesting tool to be used in epidemiological studies. However, despite of modification included in this new version, data showed in the manuscript do not support suggestions and conclusions yet, and hence, I cannot recommend publication.<br>In this work, two criteria have been used to analyse TB status of the farms: TB testing results (based on official data between 2001 and 2016), and on-animal detection of environmental MTC DNA at herd level. Detection of MTC DNA was related with three epidemiological factors (probably correlated among them): altitude of the farm, distance to woodland and the use of regional pasture. However, TB risk level does not correlated with any of the risk factors proposed. In addition, no association was detected between TB risk level (based on data from 2001-2016) and environmental MTC DNA detection at herd level.<br>->Thank you very much for offering us a sincere and critical review. It will help us improve the quality of work.<br><br>First, you are correct; there is a correlation between the use of regional pastures and the distance to forest (ANOVA p=0.021), but not with altitude (ANOVA p=0.448). This implies that the different factors that may pose a risk of exposure to mycobacteria are related and, with the data obtained in this study, it is not possible to discern which is the most determining factor.<br><br>However, as they are logically related factors, and with a potential risk of exposure to mycobacteria endowed with a biological sense, we cannot rule out any of them in our epidemiological diagnosis, and we believe that all three should be taken into account to take preventive measures to avoid new infections in cattle herds.<br><br>This new information is added in lines 245-246 and 258-262.<br><br>These results are prudently discussed in few sections of the paper. However, authors maintain in the manuscript some statements like “This study suggests that on-animal environmental DNA sampling can be a proxy for contact (or contact risk) with MTC in livestock, at the herd level”, “Our results allow us to accept the initial hypothesis: the detection of environmental DNA in cattle could contribute to risk factor identification at the cattle herd level and, always in combination with the official TB tests of individual animals, could represent a useful epidemiology tool to investigate MTC maintenance in TB hotspot areas” or “We consequently suggest that MTC DNA could be a good proxy for current infection risk. The low prevalence and incidence in the area and the apparently random detection of positive tests could indicate an environmental origin of the new cases”; which, from my point of view, are not supported by data showed in the manuscript.<br>->Thank you for the comment. In no case, our statements are intended to be overblown and, therefore, have been modified to clarify this point (lines 36-38, 249-250 and 258-262). However, we do believe that the detection of environmental DNA carried out in this study may be a good indicator of the contact risk of cattle with MTC.<br><br>To support these suggestions, it would be necessary an association between detection of MTC DNA and TB positivity at herd levels, but it has not been proven. I know that this fact can be due to the sensitivity problems of the diagnostic test used in low prevalence areas. But this fact could be also due to specificity problems of the MTC DNA detection, mainly in areas closed to woodlands where wild boar with a high percentage (26/31 (84%)) of microbiologically unconfirmed TB-like lesions (probably caused by closely related bacteria) are living.<br>Once validated, on-animal MTC DNA detection would be an useful tool to be implemented in low-prevalence areas like the studied one. However, in order to assess

Powered by Editorial Manager® and ProduXion Manager® from Aries Systems Corporation

	the association between environmental MTC DNA detection and the risk of TB appearance at herd level (based on official diagnosis method), areas with higher prevalence of TB could be better scenarios, allowing to standardize the technique (e.g. establishing percentage or number of animals tested to detect MTC DNA at herd level) and to find out more robust conclusions about the interprtation of this technique.<br>->Thank you for the comment. It is true that, a priori, we thought that the detection of environmental DNA could be directly related to the positivity of official tests. However, for biological reasons, the risk of contact with environmental MTC will always be higher than the risk of infection, and we hypothesize that, in areas of low incidence such as the one studied, the detection of environmental DNA from the areas used by livestock would make it easier to establish risk factors than the official tests themselves, which, having such a low and diffuse positivity rate, make it difficult to stablish statistical conclusions.<br><br>In addition, we have also planned to carry out new analyzes based on the official tests subsequent to this study, but, because management actions were implemented to try to reduce the prevalence in the area, the epidemiological scenario changed, and therefore the conclusions could be incorrect.<br><br><br>Reviewer #3: Jordi MARTINEZ-GUIJOSA and collaborators are reporting upon the detection of Mycobacterium tuberculosis complex (MTC) mycobacteria in herds and wild fauna in one region of Spain, where bovine tuberculosis remains of concern.<br><br>The topic is of prime interest, yet methods used to address the question are very confusing so that this reviewer had difficulties to draw any firm conclusions from the partial data herein reported.<br><br>In details:<br><br>1. The tentative integrated approach by the authors (observation of the geography, hygrometry, flora, fauna, etc…) is appropriate and very interesting, but why not sample soil which has been shown to preserve MTC mycobacteria for long, hence a potential source of contamination for the wild and farmed animals? It is a major drawback in the interpretation of reported data; and probably a missed opportunity to better understand the puzzling epidemiology of bovine tuberculosis in that region.<br>->Thank you for the comment. Yes, it is a potential weakness in our study. However, we want to clarify our point of view in this regard.<br><br>Firstly, it should be noted that the objective of this work is to test a novel on-cattle environmental DNA detection methodology. Our hypothesis is that by sampling the environmental DNA deposited on the animals, the environmental sampling is maximized by detecting the mycobacteria present in the cattle herds, which would be a proxy for the mycobacteria present in the places where they have been for the last days or weeks, contributing to risk factor identification at the herd level.<br><br>In addition, when sampling soil (presumably from a point with a potential risk of transmission of the pathogen, such as water or food), it is very difficult to sample it in an integral way (for example, the entire bottom of a water trough or all the banks of a pond), and nor do we make sure to sample the specific risk points that animals use in their normal behavior (it is extensive mountain livestock where animals are poorly managed when they are grazing freely). Through on-cattle environmental DNA detection, we expect that specificity will be lost regarding which specific places are contributing to the transmission of the pathogen, but it allows us to gain sensitivity in terms that we increase the number of relevant points sampled (potentially all recently visited by the herd) at once.<br><br>However, we assume that with soil sampling, we would have obtained additional information that would have allowed us to contrast it with our data, thus allowing a more complete view of the epidemiological scenario of the studied region and allowing us to obtain better conclusions. Therefore, we included this information in the “Discussion” section (lines 269-275) to let the reader know that we consider this weakness in our study.

Powered by Editorial Manager® and ProduXion Manager® from Aries Systems Corporation

2. The methods used to assess the presence (absence) of MTC in animals are disparate, not all tested animals have been tested with the same methods and in fact, very few animals have been really tested.

3. Among the methods used to assess the presence of MTC in animals, only culture is appropriate, catching living mycobacteria which are presumably the only ones of interest in the natural history of bovine tuberculosis. In fact, only 57 animals have been tested by culture, and only dead animals have been tested. Why not test feces which are a valuable source for MTC DNA and viable mycobacteria ?
->Thank you for the comments 2 and 3. The same method is always used to detect environmental MTC DNA, specifically, this method is described for the first time in this work and represents a new potential tool to help control and eradicate pathogens shared between wildlife and livestock. This methodology is used to infer the risk of contact with environmental MTC DNA at herd level, so it is not intended to establish the presence (absence) of MTC in each animal, and it is not necessary to sample all animals for this purpose.

Additionally, regarding the methods used in official diagnostic tests at the individual level (each cattle is tested, first by intradermal skin test, in some cases IFN-γ is required, and finally they confirm suspicious tests by culture once slaughtered), we, as scientists, cannot intervene in the official methodologies and protocols, and we can only limit ourselves to use the official data gratefully, which we also consider rigorous and reliable.

Finally, we did not tested feces because our goal was not to diagnose specific animals, but to try to infer the risk of contact of a herd with mycobacteria through dust deposited on the animals belonging to this herd. This dust comes from places that these cattle have visited naturally throughout their usual pasture behavior.

4. Methods of interest must be detailed for the reader to interpret data:

a. PCR: Negative controls? Internal DNA extraction control ? calibration curve ? Cts values ?
->Thank you for the comment. In relation to the controls included in this protocol, positive (M. bovis DNA) and negative (distilled water) controls were included for the PCR step. A sentence is included in the manuscript (lines 166): “Positive (M. bovis strain) and negative PCR controls (distilled water) were also included.”
In relation to the calibration curve, this information is not included in the manuscript since an additional manuscript is ready to be sent. To the reviewer´s knowledge, the PCR was optimized using serial dilutions of a M. bovis DNA. The range of Ct values spanned from an average of 18.83 cycles (S.D. = 0.09) at 6.5 ng/reaction to 39.18 cycles (S.D. = 0.27) at 6.5 fg/reaction. The dynamic range of the reaction spanned from 6.5 ng/reaction to 650 fg/reaction, with a R2 of 0.999 and an overall efficiency of 100%.
During the extraction step, an additional negative sample (sponge with the surfactant liquid) was included.

b. Culture: culture medium? temperature and atmosphere of incubation ? identification of colonies ?
->Thank you for the comment. The references related to the culture protocol have been modified and changed to a recent one where the conditions of decontamination, culture media and confirmation of MTBC by PCR were included. A brief description was also added to the manuscript (lines 110-115): “Briefly, approximately 2g of tissue sample was homogenized and decontaminated with an equal volume of 0.75% (w/v) hexadecyl pyridinium chloride solution in agitation during 30 minutes. After centrifugation, pellets were collected with swabs and cultured in Löwenstein-Jensen with sodium pyruvate and Coletsos media (Difco, Spain) at 37ºC up to 3 months. Culture was considered positive when isolates were identified as MTBC by conventional PCR (Wilton and Cousins, 1992) and /or DVR-spoligotyping ((Kamerbeek et al., 1997)”
https://www.ncbi.nlm.nih.gov/pubmed/1282431

Powered by Editorial Manager® and ProduXion Manager® from Aries Systems Corporation

	https://www.ncbi.nlm.nih.gov/pmc/articles/PMC229700/<br><br>Minor remarks:<br>1. Epidemiological diagnosis: this new concept is very unclear for the reviewer. In the opinion of the reviewer, the authors made a (limited) diagnosis of bovine tuberculosis, using three different methods. Interpretation of these limited diagnosis data in terms of epidemiology would require a large sample of tested animals, and integration of the sensitivity / specificity values of each one of the three tests, in calculations.<br>->Thank you. Your comment has shown us that we do not make clear the concept of epidemiological diagnosis (ED).<br>First of all, we want to clarify that we are not only referring to the characterization of the infection in the studied region, but rather we are referring to the study and analysis of the distribution, patterns and risk factors of health and disease conditions in defined populations of the studied region (at the epidemiological level). In this sense, we consider that the novel tool used in this work can help to risk factor identification at the herd level. This point has been clarified in lines70-78: "An ED consists in identifying the species involved and their TB status, along with their distribution, abundance and management within the study area. In this specific case, we have generated information on wildlife and livestock numbers and their MTC infection status, carried out interviews with farmers and used participatory Geographic Information System (GIS) [19] to investigate farm-related risk factors for the incidence of TB in cattle. The innovative nature of this study lies in the use of environmental DNA detection as a proxy for exposure risk at the farm (herd) level. Environmental DNA detection is a suitable tool by which to detect contamination with MTC in waterholes and other substrates [20–22]. We hypothesized that the detection of environmental DNA in cattle herds would contribute to risk factor identification at the herd level."<br><br>2. Line 75: Is detection of "environmental" MTC DNA really innovative?<br>->Thank you for the comment. Actually, the detection of environmental MTC DNA is not innovative, what is innovative is the tool used (the pre-hydrated sponges) and the sampling protocol, based on indirect sampling of the environment through sampling the animals that have used the resources in that environment.<br><br>3. Line 134: UCLM ?<br>->Thank you. Univesidad de Castilla la Mancha (Castilla la Mancha University)
**Additional Information:**	
**Question**	**Response**
**Financial Disclosure**<br><br>Enter a financial disclosure statement that describes the sources of funding for the work included in this submission. Review the submission guidelines for detailed requirements. View published research articles from *PLOS ONE* for specific examples.<br><br>This statement is required for submission and **will appear in the published article** if the submission is accepted. Please make sure it is accurate.	This work was supported by the Programa de Tecnologías Avanzadas en Vigilancia Sanitaria (TAVS) from the Comunidad de Madrid (ref. S2013/ABI-2747). JMG holds a FPI pre-doctoral scholarship (BES-2015- 072206), funded by MINECO. This is a contribution to MINECO grant CGL2017-89866 WildDriver and EU-FEDER.<br><br>The funders had no role in study design, data collection and analysis, decision to publish, or preparation of the manuscript.

**Unfunded studies**<br>Enter: *The author(s) received no specific funding for this work.*<br><br>**Funded studies**<br>Enter a statement with the following details:<br>• Initials of the authors who received each award<br>• Grant numbers awarded to each author<br>• The full name of each funder<br>• URL of each funder website<br>• Did the sponsors or funders play any role in the study design, data collection and analysis, decision to publish, or preparation of the manuscript?<br>• **NO** - Include this sentence at the end of your statement: *The funders had no role in study design, data collection and analysis, decision to publish, or preparation of the manuscript.*<br>• **YES** - Specify the role(s) played.<br><br>* typeset	
**Competing Interests**<br><br>Use the instructions below to enter a competing interest statement for this submission. On behalf of all authors, disclose any competing interests that could be perceived to bias this work—acknowledging all financial support and any other relevant financial or non-financial competing interests.<br><br>This statement **will appear in the published article** if the submission is accepted. Please make sure it is accurate. View published research articles from *PLOS ONE* for specific examples.	I have read the journal's policy and the authors of this manuscript have the following competing interests: MB and SABIOTEC are consultants and offer risk assessment at the wildlife-livestock interface. This does not alter our adherence to PLOS ONE policies on sharing data and materials.

Powered by Editorial Manager® and ProduXion Manager® from Aries Systems Corporation

**NO authors have competing interests**<br><br>Enter: *The authors have declared that no competing interests exist.*<br><br>**Authors with competing interests**<br><br>Enter competing interest details beginning with this statement:<br><br>*I have read the journal's policy and the authors of this manuscript have the following competing interests: [insert competing interests here]*<br><br>* typeset	
**Ethics Statement**<br><br>Enter an ethics statement for this submission. This statement is required if the study involved:<br><br>• Human participants<br>• Human specimens or tissue<br>• Vertebrate animals or cephalopods<br>• Vertebrate embryos or tissues<br>• Field research<br><br>Write "N/A" if the submission does not require an ethics statement.<br><br>General guidance is provided below. Consult the submission guidelines for detailed instructions. **Make sure that all information entered here is included in the Methods section of the manuscript.**	The UCLM research ethics committee granted a formal waiver of ethics approval since only routine veterinary care was involved in this study.

Powered by Editorial Manager® and ProduXion Manager® from Aries Systems Corporation

**Format for specific study types**<br><br>**Human Subject Research (involving human participants and/or tissue)**<br>• Give the name of the institutional review board or ethics committee that approved the study<br>• Include the approval number and/or a statement indicating approval of this research<br>• Indicate the form of consent obtained (written/oral) or the reason that consent was not obtained (e.g. the data were analyzed anonymously)<br><br>**Animal Research (involving vertebrate animals, embryos or tissues)**<br>• Provide the name of the Institutional Animal Care and Use Committee (IACUC) or other relevant ethics board that reviewed the study protocol, and indicate whether they approved this research or granted a formal waiver of ethical approval<br>• Include an approval number if one was obtained<br>• If the study involved *non-human primates*, add *additional details* about animal welfare and steps taken to ameliorate suffering<br>• If anesthesia, euthanasia, or any kind of animal sacrifice is part of the study, include briefly which substances and/or methods were applied<br><br>**Field Research**<br><br>Include the following details if this study involves the collection of plant, animal, or other materials from a natural setting:<br>• Field permit number<br>• Name of the institution or relevant body that granted permission	
**Data Availability**<br><br>Authors are required to make all data underlying the findings described fully available, without restriction, and from the time of publication. PLOS allows rare exceptions to address legal and ethical concerns. See the PLOS Data Policy and FAQ for detailed information.	Yes - all data are fully available without restriction

A Data Availability Statement describing where the data can be found is required at submission. Your answers to this question constitute the Data Availability Statement and **will be published in the article**, if accepted.<br><br>**Important:** Stating 'data available on request from the author' is not sufficient. If your data are only available upon request, select 'No' for the first question and explain your exceptional situation in the text box.<br><br>Do the authors confirm that all data underlying the findings described in their manuscript are fully available without restriction?	
**Describe where the data may be found in full sentences. If you are copying our sample text, replace any instances of XXX with the appropriate details.**<br><br>• If the data are **held or will be held in a public repository**, include URLs, accession numbers or DOIs. If this information will only be available after acceptance, indicate this by ticking the box below. For example: *All XXX files are available from the XXX database (accession number(s) XXX, XXX.*).<br>• If the data are all contained **within the manuscript and/or Supporting Information files**, enter the following: *All relevant data are within the manuscript and its Supporting Information files.*<br>• If neither of these applies but you are able to provide **details of access elsewhere**, with or without limitations, please do so. For example:<br><br>*Data cannot be shared publicly because of [XXX]. Data are available from the XXX Institutional Data Access / Ethics Committee (contact via XXX) for researchers who meet the criteria for access to confidential data.*<br><br>*The data underlying the results presented in the study are available from (include the name of the third party*	The datasets generated for this study can be found in the Mendeley Data repository (Martinez-Guijosa, Jordi (2019), "DB Environmental DNA Navarra", Mendeley Data, v1. http://dx.doi.org/10.17632/2xhxw55wny.1). Molecular epidemiology data is also available in mycoDB.es.

and contact information or URL).<br>• This text is appropriate if the data are owned by a third party and authors do not have permission to share the data.<br><br>* typeset	
Additional data availability information:	

Powered by Editorial Manager® and ProduXion Manager® from Aries Systems Corporation

Ciudad Real, March 29th 2020

PONE-D-19-20068

Environmental DNA points at shared pastures and proximity to woodlands as tuberculosis risk factors for cattle in multi-host settings

Dear Editor,

First of all, we want to wish all the members of PLOS ONE and its collaborators the best future in these troubled times. Then, we would like to thank you very much for giving us the chance to deal with the methodological issues remarked by the reviewers. We have amended the revised manuscript following all suggestions, and we tried to answer to all reviewer comments in the most complete and concise way. We also want to thank some of the reviewers for their clear agreement with our previous responses, and on the other hand, for their strong interest in finding our weak points. This makes us improve and increase the quality of our work.

Additionally, to enhance the reproducibility of our results, we deposit our laboratory protocols in the following link:
https://www.protocols.io/private/8B25CFCA69DB11EAB05E0242AC110002

For now, it is private, and its deposit will be confirmed once the article has been accepted.

I look forward to hearing from you. Yours faithfully,

Jordi Martínez-Guijosa

SaBio (Health and Biotechnology), IREC (National Game and Wildlife Research Institute; CSICUCLM-JCCM), Ciudad Real, Spain.

Manuscript Click here to access/download;Manuscript;Manuscript.docx

Environmental DNA points at shared pastures and proximity to woodlands as tuberculosis risk factors for cattle in multi-host settings

Jordi Martínez-Guijosa[1¶*], Beatriz Romero[2¶], José Antonio Infantes-Lorenzo[2,3], Elena Díez[4], Mariana Boadella[5], Ana Balseiro[6], Miguel Veiga[1], David Navarro[4], Inmaculada Moreno[7], Javier Ferreres[5], Mercedes Domínguez[7], Cesar Fernández[4], Lucas Domínguez[2,3], Christian Gortázar[1]

[1] SaBio Group, IREC (UCLM-CSIC-JCCM), Ciudad Real, Spain

[2] VISAVET Health Surveillance Centre, Complutense University of Madrid, Madrid, Spain

[3] Faculty of Veterinary Medicine, Department of Animal Health, Complutense University of Madrid, Madrid, Spain

[4] Livestock Service, Department of Rural Development, Environment and Local Administration, Government of Navarra, Pamplona, Navarra, Spain

[5] SABIOTEC, Edificio Polivalente UCLM, Ciudad Real, Spain

[6] SERIDA, Regional Service for Agrifood Research and Development, La Olla, Deva, Gijón, Asturias, Spain

[7] Area of Immunology, Microbial and Immunogenetic Immunology Unit, Instituto de Salud Carlos III, Majadahonda, Madrid, Spain

* Corresponding author

E-mail: jordi.m.guijosa@gmail.com (JMG)

[¶]These authors contributed equally to this work.

Abstract

Attaining and maintaining the Official Tuberculosis Free status continues to be a challenge when several domestic and wild hosts contribute to the maintenance of the *Mycobacterium tuberculosis* complex (MTC). Local tuberculosis hotspots are sometimes identified in cattle in low-prevalence regions. We have, therefore, studied one such a hotspot in depth in order to produce an epidemiological diagnosis. Host population size and MTC prevalence were estimated in selected wildlife and in livestock, while on-cattle environmental DNA detection was additionally used as a proxy for exposure risk at the farm (herd) level. Positive skin test reactors were found on 16 of the 24 cattle farms studied in the period 2012-2016. Although all goats tested negative to the skin test during this period, MTC was confirmed in four sheep at slaughter, thus indicating an unknown infection prevalence in this host species. With regard to wildlife, wild boar (*Sus scrofa*) MTC infection prevalence based on culture was 8.8%, and the only road-killed badger (*Meles meles*) submitted for culture tested positive. Two criteria were employed to divide the cattle farms into higher or lower risk: tuberculosis testing results and environmental DNA detection. Environmental MTC DNA detection yielded significant differences regarding "use of regional pastures" and "proximity to woodland". This study suggests that on-animal environmental DNA sampling can help in assessing contact risk with MTC in livestock, at the herd level. This tool opens up new avenues for epidemiological research in complex, multi-host settings.

Introduction

Animal tuberculosis (TB) is a multi-host infection caused by *Mycobacterium bovis* and closely related members of the *Mycobacterium tuberculosis* complex (MTC). Several EU-member states, such as France, Germany or Poland, are officially TB-free (OTF), signifying that less than 0.1% of their cattle herds are still infected. For a Member State or region to achieve OTF status as defined in Council

Directive 64/432/EEC, at least 99.9% of the cattle herds within it must have been or remained OTF for at least six consecutive years. Eradication would represent the elimination of the infection. However, achieving eradication or attaining and maintaining the OTF status continues to be a challenge when several domestic and wild hosts contribute to MTC maintenance [1].

There is still a high prevalence of TB in the south-western quarter of the Iberian Peninsula, where over 5% of herds of cattle are infected [2], the infection is present in other livestock [3–5], and there is an extremely high prevalence in wildlife, e.g. over 50% in Eurasian wild boar (*Sus scrofa*) and over 10% in red deer (*Cervus elaphus*) [6]. In other regions, however, there is a very low prevalence of TB in cattle, and it is limited in wildlife [7]. Moreover, the island regions are almost TB-free, partly owing to the absence of wildlife reservoirs [8].

There is, in fact, a low prevalence of TB in the cattle in most of the northern regions of the Spanish mainland, which are characterized by an Atlantic climate (Herd prevalence between 0.05 and 0.73, in Galicia and Aragón, respectively; year 2016 [2]). In the same regions, no TB has been detected in red deer, despite annual sampling, and TB prevalence remains relatively low in wild boar (<10%) [7,9]. European badgers (*Meles meles*) are also suitable MTC maintenance hosts, not only in the British Isles [10] but also in parts of continental Europe [11]. With regard to the Iberian Peninsula, badgers are more abundant in Atlantic than in Mediterranean regions [12], where their prevalence is reported to be around 12% [13]. Both badgers and wild boar could, therefore, contribute to MTC maintenance in Atlantic habitats in northern Spain. Roe deer (*Capreolus capreolus*), is an abundant cervid in northern Spain [14], and despite cases of infected roe deer have been evidenced, it does not appear to be a significant TB reservoir [15,16]. Sheep [5] and goats [7] may also contribute toward increasing the complexity of the MTC host network on a local basis.

Local TB hotspots, i.e., areas of elevated disease incidence or prevalence or a geographic cluster of cases [17], are sometimes identified in these low-prevalence Atlantic settings. We have, therefore,

studied one of these hotspots in depth in order to produce an epidemiological diagnosis (ED). This ED is a step proposed by the Spanish Wildlife TB Control Plan (PATUBES,[18]), prior to any intervention. An ED consists in identifying the species involved and their TB status, along with their distribution, abundance and management within the study area. In this specific case, we have generated information on wildlife and livestock numbers and their MTC infection status, carried out interviews with farmers and used participatory Geographic Information System (GIS) [19] to investigate farm-related risk factors for the incidence of TB in cattle. The innovative nature of this study lies in the use of environmental DNA detection as a proxy for exposure risk at the farm (herd) level. Environmental DNA detection is a suitable tool by which to detect contamination with MTC in waterholes and other substrates [20–22]. We hypothesized that the detection of environmental DNA in cattle herds would contribute to risk factor identification at the herd level.

Materials and methods

Study area

The study area is located in the north of the Iberian Peninsula, between Atlantic Spain and the pre-Pyrenean north plateau (centroid coordinates, utm wgs84: 592425, 4743194), to the northwest of Pamplona, in the region of Navarra. This area is a pre-Pyrenean basin that has a marked altitudinal gradient and several rivers and tributaries that form abundant ravines, and is characterized by the presence of the Sierra Urbasa-Andía to the west (a mountainous Natural Park). It occupies an area of approximately 100 Km^2, with an average altitude of 830 meters (range: 384-1260m) and an average annual temperature of 8º - 12ºC. The livestock management system of the area is largely determined by a marked seasonal variation in temperatures and rainfall. The maximum monthly precipitations are collected in December and the minimum in July. The dominant vegetation comprises Portuguese oak (*Quercus faginea*) and holm oak (*Quercus rotundifolia*) forests, and those of beech (*Fagus sylvatica*)

in higher or shady areas. The forests occupy 45% of the area, and cover two thirds of the total surface if we consider scrublands. The remaining third is occupied by pastures, meadows and arable crops.

The land use in the study area is principally determined by the type of property, of which there are three different categories: private land, municipal land and regional land [23]. The private land, which is owned by farmers, is where the barns and warehouses are located. Each municipality has communal land to which every inhabitant of that village has access for livestock grazing and watering. The regional land (Urbasa-Andía Natural Park) has similar rights to those of the municipal land, with the difference that their use extends to all the farmers in the region.

Wildlife densities and TB status

Wild boar densities were assessed using two means. On the one hand, we used three 4x4 photo-trapping grids (16 cameras each, with a distance of 1km between cameras; S1 Fig), installed from September 5 to October 12 (2016), to estimate densities on the basis of an algorithm described previously [24], based on the random detection likelihood of unmarked individuals. We assumed a mean daily speed of 0.115 m/s (own data), a detection radius of 10m and a detection angle of 35º. On the other hand, we used the 2015/16 hunting data to assess the number of wild boar observed per square km. Badger densities were estimated using the same camera-trapping grids and camera specifications and a mean daily speed of 0.021 m/s. This speed was based on data obtained from radio-tracked badgers in Asturias, Spain. Fifty seven hunter-harvested wild boar and one road-killed badger were inspected for visible TB-compatible lesions [25] and submitted for bacteriological culture and molecular identification in a BSL3 laboratory as described previously [26]. Briefly, approximately 2g of tissue sample was homogenized and decontaminated with an equal volume of 0.75% (w/v) hexadecyl pyridinium chloride solution in agitation during 30 minutes. After centrifugation, pellets were collected with swabs and cultured in Löwenstein-Jensen with sodium pyruvate and Coletsos media (Difco,

Spain) at 37ºC up to 3 months. Culture was considered positive when isolates were identified as MTBC by conventional PCR [27] and /or DVR-spoligotyping [28].

Livestock herds, pasture management and TB status

The study area includes 36 cattle herds with 3,100 cattle in total, 3 herds of goats (a total of 309 goats) and 17 flocks of sheep totaling 4,786 sheep. No pigs are present. With regard to the herds of cattle, 12 were not studied because they were feedlots, because of technical restrictions or owing to their very small size (<10 cattle). The 24 herds selected for the study added 2,786 cattle (89.87% of the total cattle in the study area). All cattle and goats are tested for TB on an annual basis by means of the official single intradermal tuberculin test according to the Spanish Eradication Program of Bovine Tuberculosis [2], and data for the period 2012-2016 was, therefore, kindly provided by the Government of Navarra.

The grazing system is complex, since herds belonging to different owners share pastures and water points. The farms that use the regional pastures (14 of the 24 farms sampled) take their livestock to this land in spring (May) in order to take advantage of these pastures before moving to municipal and private land in summer (August). Those farmers who do not use the regional pastures use the municipal pastures in their place between May and November (9 of the 24 farms sampled). In winter, when it begins to snow, the cattle are moved to stables and fed with fodder or on pastures close to the farm building. Only one farm is limited to using exclusively private land and does not share pastures with other farmers at any time.

All the work performed on the farms was carried out between September 2016 and January 2017, following the recommendations and protocols described in the Spanish Eradication Program of Bovine Tuberculosis [2]. Additionally, the UCLM research ethics committee granted a formal waiver of ethics approval since only routine veterinary care was involved in this study.

On-farm risk assessment

Only two categorical variables (yes/no) were studied, namely the use of regional pastures and the use of municipal pastures. Table 1 lists the quantitative variables considered per cattle farm, and the source of the information.

Table 1. Quantitative variables analyzed, origin of the data, Z statistic and P value (Mann-Whitney's U test) for each of the two criteria defined in this study: Outbreak frequency based on TB testing results and environmental MTC DNA positivity.

		**TB testing** (>1 year positive)		**MTC DNA** (detected)	
**Variable**	**Data source**	**Z**	**P**	**Z**	**P**
Total N of cattle	Official data	1.92	0.056	-1.79	0.078
Number of heifers	Official data	1.80	0.076	-1.30	0.19
N of cattle groups for pasture	Farmer interview	0.64	0.55	0.40	0.69
N of imported cattle (5 years)	Official data	0.02	0.98	-0.73	0.47
N of import movements (5 years)	Official data	0.53	0.59	0.35	0.75
Total land used	Farmer interview	-1.08	0.31	-1.12	0.27
Total own land	Farmer interview	0.82	0.41	0.39	0.72
Total own pastures	Farmer interview	-0.36	0.72	0.71	0.50
Total own woodland	Farmer interview	-0.27	0.82	-0.92	0.42
N own goats	Farmer interview	1.64	0.44	0.24	0.89
N own sheep	Farmer interview	0.65	0.73	-0.51	0.78
N own horses	Farmer interview	1.28	0.20	0.53	0.65
N users' municipal pastures	Farmer interview	-1.23	0.23	-1.79	0.088

**Farm altitude a.s.l.**	GIS	0.96	0.35	2.42	**0.014**
Farm distance to village	GIS	-1.35	0.18	-1.68	0.10
**Farm distance to woodland**	GIS	1.35	0.18	2.68	**0.006**
N of identified risk points	Farm visit	-0.26	0.81	-1.27	0.21
Freq. sees wild boar	Farmer interview	-0.02	0.98	0	1
Freq. sees roe deer	Farmer interview	-0.24	0.83	-0.56	0.60
Freq. sees badger	Farmer interview	-0.08	0.94	1.34	0.26

Significant results are shown in bold type. a.s.l = at sea level.

Environmental DNA

During January 2017, samples of environmental DNA were taken from cattle from 24 farms. A dry sponge (3M™ Dry-Sponge; 3M-España, Madrid) was pre-hydrated with 15 ml of an isotonic surfactant liquid. Alternatively, 15 ml of sterile distilled water can be used. On each farm, two sponges were scrubbed ten times on the scapula regions of two randomly chosen cattle (one sponge by animal). The scapula region, and no other anatomical region of the animal, was chosen because it is the most accessible area during cattle handling which is relatively close to the brisket, one of the preferred areas for sampling pathogens on cattle hides [29]. In addition, it is a large and quasi-horizontal area, where dust particles can easily deposit. In the laboratory, the sponge was hydrated with 10 ml of the same liquid and centrifuged at 3,500 *g* for 10 minutes. All the steps until recovery of DNA were performed under safety conditions in a BSL3 area.

The pellet was purified as previously described, including mechanical disruption with beads using the fast-prep equipment (before and after the enzymatic lysis) [26]. A real time PCR with which to detect *IS6110* was used to detect MTC in the samples, using the primers (forward 5’-GGTAGCAGACCTCACCTATGTGT-3’; reverse 5’-AGGCGTCGGTGACAAAGG-3’) and probe

(5' FAM-CACGTAGGCGAACCC-MGB-NFQ 3') described previously [30]. The PCR was performed using the QuantiFast Pathogen +IC Kit, according to the manufacturer's instructions (Qiagen, Hilden, Germany), with a final concentration of 2.5 µM for each primer, 1.25 µM for the probe, and 5 µl of the extracted DNA. All PCR reactions were carried out in a CFX96 TouchTM Real-Time PCR Detection System (Bio-Rad, Hercules, CA, USA) according to the following cycling conditions: 95ºC for 5 minutes followed by 45 2-step cycles at 95ºC for 15 seconds and 60ºC for 30 seconds. Positive (*M. bovis* strain) and negative PCR controls (distilled water) were also included. A conventional PCR was also used to discard the presence of *Mycobacterium avium* subspecies [27]. The analytical specificity of the PCR was tested in 112 DNA samples from pure cultures from 28 *Mycobacterium* species (not belonging to MTC), 5 *Streptomyces sp.*, and one *Lactobacillus brevis* strain. Cross-reaction was detected in few *M. avium subsp. hominissuis* isolates.

Statistics

We decided to use only non-parametric statistics owing to the relatively low sample size (n=24 cattle farms). The Chi square or Fisher's test were used (when appropriate) for categorical data, while Mann-Whitney's U test was employed for numerical data.

Results

The local MTC maintenance host community

The local host community is composed of 3,100 cattle, 309 goats and 4,786 sheep as regards livestock; and between 232 and 431 wild boar (mean density 2.3 to 4.3/km^2 based on photo-trapping and hunting data, respectively) and 91 badgers (photo-trapping; mean density 0.9/km^2), as regards suitable wildlife MTC host species.

Of the 24 cattle farms sampled, eight were negative to the skin test during 2012-2016. The remaining 16 farms had reactors to the skin test with an average of 28.6 positive skin-test animals/year, and none of the reactor farms was consistently positive in all five years (the largest series of consecutive years that a farm was positive was three and four, and this occurred on only two of the 16 reactor farms). This produced a chessboard-like figure of TB incidence in cattle in the study area (S1 Table). All the goats tested during the last five years had obtained negative results to intradermal skin test. However, TB compatible lesions were detected in four sheep as part of the slaughterhouse surveillance, revealing an unknown infection prevalence in this host species.

With regard to wildlife, we sampled 57 hunter-harvested wild boar. The TB-compatible lesion prevalence was 54% (31/57) and the infection prevalence confirmed by culture was 8.8% (5/57). Only one road-killed badger was submitted for culture and was positive. All cattle (N= 7), sheep (N= 4), wild boar (N= 5) and badger (N= 1) isolates obtained in the study were identified as *M. bovis* and were classified with SB0134 spoligotype (according to the Mbovis.org database).

Farm TB status and environmental DNA

Based on the results of the annual intradermal test, the farms were classified as farms with a higher risk, those that were positive to TB for at least two years (consecutive or otherwise) (n=10), and farms with a lower risk, i.e., those with reactors in one year or without reactors (n = 14), in the period of study (2012-2016). Environmental MTC DNA was detected in cattle from 12 of the 24 sampled farms (50% positive farms; 15 positive samples out of 48, 31.3% positive samples). There was no significant association between the higher risk area and the detection of environmental DNA (Fisher's test, P = 0.982). *Mycobacterium avium* subspecies was only detected in one sample from a farm where the additional sponge was only positive to MTC.

Regional pastures were used by 10 of the 12 MTC DNA positive farms but only by 3/12 MTC DNA negative ones (Fisher's test, P=0.006). The use of municipal pastures was only marginally significant (Fisher's test, P=0.088). With regard to the quantitative variables, altitude and distance to woodland were significant (Mann-Whitney's U test, P=0.014 and P=0.006, respectively. Table 1), indicating that farms at higher altitude and closer to the nearest woodland edge (wildlife habitat) were at higher risk of MTC DNA detection (Fig 2). In contrast, we found no significant difference between "higher risk" and "lower risk" cattle farms as regards using regional pastures or municipal pastures (Fisher's test, P=0.9449 and P=0.704, respectively), and no differences were found for the 20 quantitative variables.

Fig 1. Shared use of communal pastures as a risk factor depending on environmental MTC DNA detection.

Fig 2. Cattle-shelter proximity to woodlands as a risk factor depending on environmental MTC DNA detection. Points represent the different farms studied. Distance expressed in meters.

Discussion

The ED carried out in this tuberculosis hotspot region evidenced that MTC is maintained in the study area owing to a multi-host community composed of domestic and wild hosts (with confirmed infection in cattle, sheep, wild boar, and badger), despite all the efforts made by the TB eradication program. Environmental sampling has been used as an additional test to detect mycobacteria on the farms and the results revealed that shared regional pastures and proximity to woodlands were risk factors.

In the study area, about 40 individual cattle become infected annually (based on 2016 official TB testing). Considering that the sensitivity of the single intradermal test is around 70% [31], it would be possible to estimate a higher number of infected cattle (around 52) in this year in the study area. There were, simultaneously, also about 20 to 38 MTC infected wild boar (at a minimum prevalence of 8.8%), plus an unknown number of infected sheep and badgers, sharing the habitat with cattle. It is consequently necessary to assume that MTC, as a paradigmatic multi-host infection, is maintained by a complex matrix of wild and domestic hosts. Any intervention should, therefore, target all possible hosts and not just cattle. Moreover, host populations are not static. The number of wild boar in the study region has, for example, tripled since 1990, and continues to increase [32,33]. Badger abundance is also known to have increased in northern Spain [34]. This and other changes in the composition of the host community, along with the changes in wildlife and livestock management and in livestock numbers, will likely modify the relative importance of the risk factors for shared infections over time.

This study suggests that on-animal environmental DNA sampling can help in assessing contact risk with MTC in livestock, at the herd level. Regional pastures can freely be used by all herds of cattle in this region, while municipal pastures are shared only with farms located in the same municipality. One risk linked to shared pastures is obviously contact with other herds of cattle. However, additional risks as regards regional (or municipal) pastures are the contact with other livestock (in this case, flocks of sheep) and the contact with wildlife. Regional pastures tend to be at higher altitudes and are more suitable for wildlife such as wild boar. In our specific study area, the regional pasture area is, moreover, a protected natural park, where wild boar hunting is restricted. The identification of communal pastures as a risk factor for cattle TB coincides with recent findings obtained from a large-scale survey in Castilla y León, Spain, in which communal pastures were also found to be a risk factor for wild boar TB [7]. In addition, farm altitude and distance to woodland proved significant (both factors are correlated with each other; Pearson's $r = 0.45$; P= 0.024). Use of regional pastures is also correlated

with distance to woodland, but not with altitude; ANOVA P= 0.021, P= 0.448, respectively). We suggest that those factors are a proxy for the risk of contact with wildlife MTC hosts such as badgers and wild boar. Epidemiological links between badgers, wild boar and cattle have already been evidenced in northern Spain [9,35] and elsewhere [36].

Our results prevent us from rejecting the initial hypothesis: the detection of environmental DNA in cattle could contribute to risk factor identification at the cattle herd level and, always in combination with the official TB tests of individual animals, could represent a useful epidemiology tool to investigate MTC maintenance in TB hotspot areas. This is of particular relevance given the difficulties involved in interpreting TB testing outcomes in low-prevalence settings [37]. While this finding might, at first glance, appear to be counter-intuitive, it can easily be explained: a positive intradermal official test reflects that cattle have previously come into contact with MTC, and the reactors are almost immediately slaughtered and hence removed from the study herds. In contrast, MTC DNA detection in a herd of cattle, while not necessarily reflecting infection, suggests current contact with an MTC-contaminated source, e.g. wildlife or the environment. The low prevalence and incidence in the area and the apparently random detection of positive tests could suggest an environmental origin of the new cases. Despite in this study it was not possible to elucidate the true origin of MTC infection in the studied cattle herds, MTC DNA detection has allowed to reveal specific risk factors on which to act through farm biosafety.

Additional research is needed to verify the usefulness of this technique in different situations where its implementation has the potential to support the economic and sanitary viability of livestock farms, such as, for example, in farms with recurrent TB cases, or after stamping out and disinfection of the farm in order to avoid reinfection from environmental sources. Additionally, we assume that soil sampling could be a very valuable complementary tool, with which we would have obtained information that would have allowed us to contrast it with our data, thus allowing a more complete

view of the epidemiological scenario of the studied region and allowing us to obtain better conclusions. Therefore, additional research is needed, integrating different tools beyond those used in this study that complement the information necessary to carry out comprehensive and useful epidemiological diagnoses.

Additional care is advised for two reasons. First, the real time PCR used to detect MTC DNA is based on the IS*6110* insertion sequence. While all efforts have been made to confirm specificity, such as discarding the presence of *Mycovacterium avium* subspecies, a small risk of cross reaction with some environmental mycobacteria or with other agents, such as certain *Streptomyces* strains, could exist. We assume this limited specificity in some scenarios although this should not invalidate our results. Moreover, *Mycobacterium microti*, a pathogen belonging to the MTC but not included among the main causal agents of bovine TB, will cause positive PCR results [38]. Fortunately, *M. microti* is very rarely reported in Spain (only five records in mycodb.es) [39].

The tool described herein, which detects MTC DNA at the herd level, opens up new avenues for epidemiological research and facilitates ED. This tool will hopefully provide additional insights into the herd-level risk factors for contact with MTC and other pathogens in a broad range of settings.

Acknowledgments

We are indebted to all the farmers who have participated in this study. We are also grateful to all the hunters and field veterinarians who diligently collaborated in sample collection. Last, but by no means least, we would also like to thank the Governments of Navarra’s Environmental Service and Forest Guard Section their full collaboration in this project.

References

1. Gortázar C, Che Amat A, O'Brien DJ. Open questions and recent advances in the control of a multi-host infectious disease: Animal tuberculosis. Mamm Rev. 2015;45: 160–175. doi:10.1111/mam.12042

2. Spanish Ministry of Agriculture and Fisheries Food and Environment. Spanish Eradication Program of Bovine Tuberculosis [Internet]. Spain; 2018. Available: https://www.mapa.gob.es/es/ganaderia/temas/sanidad-animal-higiene-ganadera/pnetb_2018_tcm30-436761.pdf

3. Aranaz A, Liébana E, Gómez-Mampaso E, Galán JC, Cousins D, Ortega A, et al. *Mycobacterium tuberculosis* subsp. *caprae* subsp. *nov.*: A taxonomic study of a new member of the Mycobacterium tuberculosis complex isolated from goats in Spain. Int J Syst Bacteriol. 1999;49: 1263–1273. doi:10.1099/00207713-49-3-1263

4. Parra A, Fernández-Llario P, Tato A, Larrasa J, García A, Alonso JM, et al. Epidemiology of *Mycobacterium bovis* infections of pigs and wild boars using a molecular approach. Vet Microbiol. 2003;97: 123–133. doi:10.1016/j.vetmic.2003.08.007

5. Muñoz-Mendoza M, Romero B, del Cerro A, Gortázar C, García-Marín JF, Menéndez S, et al. Sheep as a Potential Source of Bovine TB: Epidemiology, pathology and evaluation of diagnostic techniques. Transbound Emerg Dis. 2016;63: 635–646. doi:10.1111/tbed.12325

6. Vicente J, Barasona JA, Acevedo P, Ruiz-Fons JF, Boadella M, Diez-Delgado I, et al. Temporal trend of tuberculosis in wild ungulates from mediterranean Spain. Transbound Emerg Dis. 2013;60: 92–103. doi:10.1111/tbed.12167

7. Gortázar C, Fernández-Calle LM, Collazos-Martínez JA, Mínguez-González O, Acevedo P. Animal tuberculosis maintenance at low abundance of suitable wildlife reservoir hosts: A case

study in northern Spain. Prev Vet Med. 2017;146: 150–157. doi:10.1016/j.prevetmed.2017.08.009

8. Acevedo P, Romero B, Vicente J, Caracappa S, Galluzzo P, Marineo S, et al. Tuberculosis Epidemiology in Islands: Insularity, Hosts and Trade. PLoS One. 2013;8: e71074. doi:10.1371/journal.pone.0071074

9. Muñoz-Mendoza M, Marreros N, Boadella M, Gortázar C, Menéndez S, de Juan L, et al. Wild boar tuberculosis in Iberian Atlantic Spain: A different picture from Mediterranean habitats. BMC Vet Res. 2013;9: 176. doi:10.1186/1746-6148-9-176

10. Vial F, Johnston WT, Donnelly CA. Local cattle and badger populations affect the risk of confirmed tuberculosis in British cattle herds. PLoS One. 2011;6: e18058. doi:10.1371/journal.pone.0018058

11. Bouchez-Zacria M, Courcoul A, Jabert P, Richomme C, Durand B. Environmental determinants of the *Mycobacterium bovis* concomitant infection in cattle and badgers in France. Eur J Wildl Res. 2017;63: 74. doi:10.1007/s10344-017-1131-4

12. Acevedo P, González-Quirós P, Prieto JM, Etherington TR, Gortázar C, Balseiro A. Generalizing and transferring spatial models: A case study to predict Eurasian badger abundance in Atlantic Spain. Ecol Modell. 2014;275: 1–8. doi:10.1016/j.ecolmodel.2013.12.011

13. Balseiro A, Rodríguez O, González-Quirós P, Merediz I, Sevilla IA, Davé D, et al. Infection of Eurasian badgers (*Meles meles*) with *Mycobacterium bovis* and *Mycobacterium avium* complex in Spain. Vet J. 2011;190: e21–e25. doi:10.1016/j.tvjl.2011.04.012

14. Acevedo P, Delibes-Mateos M, Escudero MA, Vicente J, Marco J, Gortazar C. Environmental

constraints in the colonization sequence of roe deer (*Capreolus capreolus* Linnaeus, 1758) across the Iberian Mountains, Spain. J Biogeogr. 2005;32: 1671–1680. doi:10.1111/j.1365-2699.2005.01310.x

15. Lambert S, Hars J, Réveillaud E, Moyen JL, Gares H, Rambaud T, et al. Host status of wild roe deer in bovine tuberculosis endemic areas. Eur J Wildl Res. 2017;63: 15. doi:10.1007/s10344-016-1071-4

16. Balseiro A, Oleaga Á, Orusa R, Roberto S, Zoppi S, Dondo A, et al. Tuberculosis in roe deer from Spain and Italy. Vet Rec. 2009;164: 468–470. doi:10.1136/vr.164.15.468

17. Lessler J, Azman AS, McKay HS, Moore SM. Perspective piece: What is a hotspot anyway? Am J Trop Med Hyg. 2017;96: 1270–1273. doi:10.4269/ajtmh.16-0427

18. Spanish Ministry of Agriculture and Fisheries F and E. Spanish Wildlife TB Control Plan [Internet]. Spain: Spanish Ministry of Agriculture and Fisheries, Food and Environment; 2017. Available: https://www.mapa.gob.es/es/ganaderia/temas/sanidad-animal-higiene-ganadera/patubes2017_3_tcm30-378321.pdf

19. Cowie CE, Gortázar C, White PCL, Hutchings MR, Vicente J. Stakeholder opinions on the practicality of management interventions to control bovine tuberculosis. Vet J. 2015;204: 179–185. doi:10.1016/j.tvjl.2015.02.022

20. Barasona JA, Vicente J, Díez-Delgado I, Aznar J, Gortázar C, Torres MJ. Environmental Presence of Mycobacterium tuberculosis Complex in Aggregation Points at the Wildlife/Livestock Interface. Transboundary and Emerging Diseases. 2017: 1148–1158. doi:10.1111/tbed.12480

21. Santos N, Almeida V, Gortázar C, Correia-Neves M. Patterns of *Mycobacterium tuberculosis*-

complex excretion and characterization of super-shedders in naturally-infected wild boar and red deer. Vet Res. 2015;46: 129. doi:10.1186/s13567-015-0270-4

22. Barbier E, Boschiroli ML, Gueneau E, Rochelet M, Payne A, de Cruz K, et al. First molecular detection of *Mycobacterium bovis* in environmental samples from a French region with endemic bovine tuberculosis. J Appl Microbiol. 2016;120: 1193–1207. doi:10.1111/jam.13090

23. Cuadrado Iglesias M. Aprovechamientos en común de pastos y leñas. Madrid, ES: Ministerio de Agricultura, Pesca y Alimentación, Servicio de Extensión Agraria; 1980.

24. Rowcliffe JM, Field J, Turvey ST, Carbone C. Estimating animal density using camera traps without the need for individual recognition. J Appl Ecol. 2008;45: 1228–1236. doi:10.1111/j.1365-2664.2008.01473.x

25. Vicente J, Höfle U, Garrido JM, Fernández-De-Mera IG, Juste R, Barral M, et al. Wild boar and red deer display high prevalences of tuberculosis-like lesions in Spain. Vet Res. 2006;37: 107–119. doi:10.1051/vetres:2005044

26. Lorente-Leal V, Liandris E, Castellanos E, Bezos J, Domínguez L, de Juan L, et al. Validation of a real-time PCR for the detection of *Mycobacterium tuberculosis* complex members in Bovine tissue samples. Front Vet Sci. 2019;6: 61. doi:10.3389/fvets.2019.00061

27. Wilton S, Cousins D. Detection and identification of multiple mycobacterial pathogens by DNA amplification in a single tube. Genome Res. 1992;1: 269–273. doi:10.1101/gr.1.4.269

28. Kamerbeek J, Schouls L, Kolk A, Van Agterveld M, Van Soolingen D, Kuijper S, et al. Simultaneous detection and strain differentiation of *Mycobacterium tuberculosis* for diagnosis and epidemiology. J Clin Microbiol. 1997;35: 907–914. doi:10.1016/S0305-4403(02)00239-X

29. Reid CA, Small A, Avery SM, Buncic S. Presence of food-borne pathogens on cattle hides. Food Control. 2002;13: 411–415. doi:10.1016/S0956-7135(01)00050-0

30. Michelet L, de Cruz K, Karoui C, Hénault S, Boschiroli ML. *Mycobacterium microti*, un agent tuberculeux méconnu. In: Dufour Barbara, editor. Épidémiol Et Santé Anim. Maisons-Alfort Cedex, France: Journée scientifique AEEMA, 24 mars 2017; 2017. pp. 71, 129–138.

31. Bezos J, Casal C, Romero B, Schroeder B, Hardegger R, Raeber AJ, et al. Current ante-mortem techniques for diagnosis of bovine tuberculosis. Res Vet Sci. 2014;97: S44–S52. doi:10.1016/j.rvsc.2014.04.002

32. Acevedo P, Quirós-Fernández F, Casal J, Vicente J. Spatial distribution of wild boar population abundance: Basic information for spatial epidemiology and wildlife management. Ecol Indic. 2014;36: 594–600. doi:10.1016/j.ecolind.2013.09.019

33. Leránoz I, Castién E. Evolución de la población del jabalí (*Sus scrofa* L., 1758) en Navarra (N Península Ibérica). Miscel· lània Zoològica. 1996;19: 133–139.

34. Sobrino R, Acevedo P, Escudero MA, Marco J, Gortázar C. Carnivore population trends in Spanish agrosystems after the reduction in food availability due to rabbit decline by rabbit haemorrhagic disease and improved waste management. Eur J Wildl Res. 2009;55: 161–165. doi:10.1007/s10344-008-0230-7

35. Balseiro A, González-Quirós P, Rodríguez Ó, Francisca Copano M, Merediz I, de Juan L, et al. Spatial relationships between Eurasian badgers (*Meles meles*) and cattle infected with *Mycobacterium bovis* in Northern Spain. Vet J. 2013;197: 739–745. doi:10.1016/j.tvjl.2013.03.017

36. Payne A, Chappa S, Hars J, Dufour B, Gilot-Fromont E. Wildlife visits to farm facilities

assessed by camera traps in a bovine tuberculosis-infected area in France. Eur J Wildl Res. 2016;62: 33–42. doi:10.1007/s10344-015-0970-0

37. Álvarez J, Pérez A, Marqués S, Bezos J, Grau A, de la Cruz ML, et al. Risk factors associated with negative in-vivo diagnostic results in bovine tuberculosis-infected cattle in Spain. BMC Vet Res. 2014;10: 14. doi:10.1186/1746-6148-10-14

38. Barbier E. Prévalence de *Mycobacterium bovis* dans les agroécosystèmes : analyse de réservoirs environnementaux potentiels (sol, eau douce, faune du sol et faune aquatique) et traçage de la circulation de cette bactérie entre les différents compartiments [Internet]. (Doctoral dissertation). 2016. doi:tel-01476359

39. De Val BP, Sanz A, Soler M, Allepuz A, Michelet L, Boschiroli ML, et al. *Mycobacterium microti* infection in Free-Ranging Wild Boar, Spain, 2017-2019. Emerging Infectious Diseases. Centers for Disease Control and Prevention (CDC); 2019. pp. 2152–2154. doi:10.3201/eid2511.190746

Supporting information

S1 Fig. Location of the photo-trapping grids in the study area.

S1 Table. Official TB testing annual results of the sampled farms. Each column shows the positive animals (cattle) in the respective years, categorized by diagnostic technique according to the following color code: Positive Tuberculin Intradermal Reaction (red); Positive Interferon-γ Gamma Release Assay (blue); Positive Slaughter (Lesions) (yellow); Positive Culture (green). If the same animal has tested positive for more than one diagnostic test, only the color of the most restrictive technique is shown. The last column shows the environmental MTC DNA sampling results

categorized as MTC (*Mycobacterium tuberculosis* Complex), MAC (*Mycobacterium avium* Complex), *Mycobacterium* sp. (genus *Mycobacterium* not belonging to MTC or MAC) and 0 (negative or not conclusive).

Figure 1

Click here to access/download;Figure;Fig1.tif

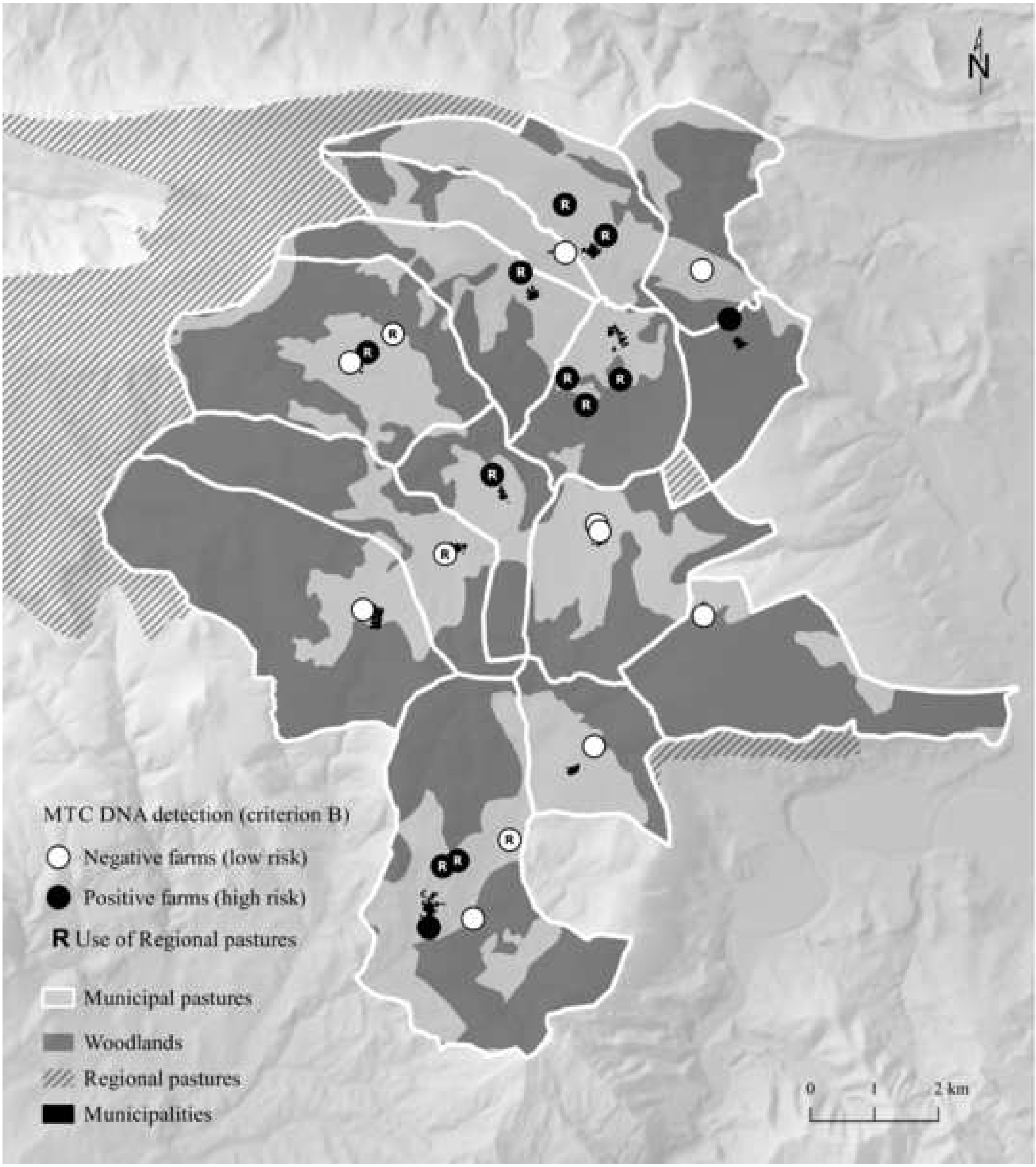

Figure 2

Click here to access/download;Figure;Fig2.tif

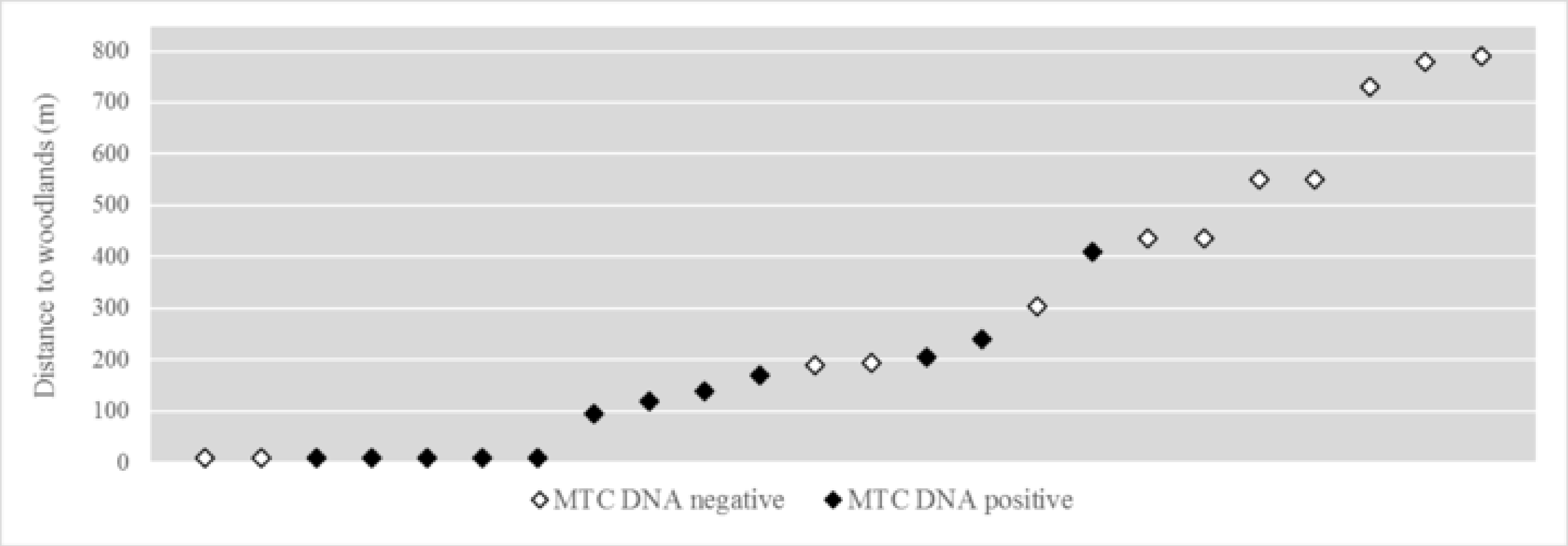

Supporting Information Figure

Click here to access/download
Supporting Information
S1 Fig.tif

Supporting Information Table

Click here to access/download
Supporting Information
S1 Table.pdf

Environmental DNA points at shared pastures and proximity to woodlands as tuberculosis risk factors for cattle in multi-host settings

Jordi Martínez-Guijosa[1¶*], Beatriz Romero[2¶], José Antonio Infantes-Lorenzo[2,3], Elena Díez[4], Mariana Boadella[5], Ana Balseiro[6], Miguel Veiga[1], David Navarro[4], Inmaculada Moreno[7], Javier Ferreres[5], Mercedes Domínguez[7], Cesar Fernández[4], Lucas Domínguez[2,3], Christian Gortázar[1]

[1] SaBio Group, IREC (UCLM-CSIC-JCCM), Ciudad Real, Spain

[2] VISAVET Health Surveillance Centre, Complutense University of Madrid, Madrid, Spain

[3] Faculty of Veterinary Medicine, Department of Animal Health, Complutense University of Madrid, Madrid, Spain

[4] Livestock Service, Department of Rural Development, Environment and Local Administration, Government of Navarra, Pamplona, Navarra, Spain

[5] SABIOTEC, Edificio Polivalente UCLM, Ciudad Real, Spain

[6] SERIDA, Regional Service for Agrifood Research and Development, La Olla, Deva, Gijón, Asturias, Spain

[7] Area of Immunology, Microbial and Immunogenetic Immunology Unit, Instituto de Salud Carlos III, Majadahonda, Madrid, Spain

* Corresponding author

E-mail: jordi.m.guijosa@gmail.com (JMG)

[¶]These authors contributed equally to this work.

Abstract

Attaining and maintaining the Official Tuberculosis Free status continues to be a challenge when several domestic and wild hosts contribute to the maintenance of the *Mycobacterium tuberculosis* complex (MTC). Local tuberculosis hotspots are sometimes identified in cattle in low-prevalence regions. We have, therefore, studied one such a hotspot in depth in order to produce an epidemiological diagnosis. Host population size and MTC prevalence were estimated in selected wildlife and in livestock, while on-cattle environmental DNA detection was additionally used as a proxy for exposure risk at the farm (herd) level. Positive skin test reactors were found on 16 of the 24 cattle farms studied in the period 2012-2016. Although all goats tested negative to the skin test during this period, MTC was confirmed in four sheep at slaughter, thus indicating an unknown infection prevalence in this host species. With regard to wildlife, wild boar (*Sus scrofa*) MTC infection prevalence based on culture was 8.8%, and the only road-killed badger (*Meles meles*) submitted for culture tested positive. Two criteria were employed to divide the cattle farms into higher or lower risk: tuberculosis testing results and environmental DNA detection. Environmental MTC DNA detection yielded significant differences regarding "use of regional pastures" and "proximity to woodland". This study suggests that on-animal environmental DNA sampling can ~~be a proxy for~~help in assessing ~~contact (or~~ contact risk~~)~~ with MTC in livestock, at the herd level. This ~~new~~ tool opens up new avenues for epidemiological research ~~and facilitates epidemiological diagnoses~~ in complex, multi-host settings.

Introduction

Animal tuberculosis (TB) is a multi-host infection caused by *Mycobacterium bovis* and closely related members of the *Mycobacterium tuberculosis* complex (MTC). Several EU-member states, such as France, Germany or Poland, are officially TB-free (OTF), signifying that less than 0.1% of their

~~herds of~~ cattle herds are still infected. ~~In order f~~For a Member State or region to achieve OTF status as defined in Council Directive 64/432/EEC, at least 99.9% of the ~~herds of~~ cattle herds within it must have been or remained OTF for at least six consecutive years. Eradication would represent the elimination of the infection. However, achieving eradication or attaining and maintaining the OTF status continues to be a challenge when several domestic and wild hosts contribute to MTC maintenance [1].

There is still a high prevalence of TB in the south-western quarter of the Iberian Peninsula, where over 5% of herds of cattle are infected [2], the infection is present in other livestock [3–5], and there is an extremely high prevalence in wildlife, e.g. over 50% in Eurasian wild boar (*Sus scrofa*) and over 10% in red deer (*Cervus elaphus*) [6]. In other regions, however, there is a very low prevalence of TB in cattle, and it is limited in wildlife [7]. Moreover, the island regions are almost TB-free, partly owing to the absence of wildlife reservoirs [8].

There is, in fact, a low prevalence of TB in the cattle in most of the northern regions of the Spanish mainland, which are characterized by an Atlantic climate (H~~h~~erd prevalence between 0.05 and 0.73, in Galicia and Aragón, respectively;~~.~~ y~~Y~~ear 2016 [2]). In the same regions, no TB has been detected in red deer, despite annual sampling, and TB prevalence remains relatively low in wild boar (<10%) [7,9]. European badgers (*Meles meles*) are also suitable MTC maintenance hosts, not only in the British Isles [10] but also in parts of continental Europe [11]. With regard to the Iberian Peninsula, badgers are more abundant in Atlantic than in Mediterranean regions [12], where their prevalence is reported to be around 12% [13]. Both badgers and wild boar could, therefore, contribute to MTC maintenance in Atlantic habitats in northern Spain. Roe deer (*Capreolus capreolus*), is an abundant cervid in northern Spain [14], and despite cases of infected roe deer have been evidenced, it does not appear to be a significant TB reservoir [15,16]. Sheep [5] and goats [7] may also contribute toward increasing the complexity of the MTC host network on a local basis.

Local TB hotspots, i.e., areas of elevated disease incidence or prevalence or a geographic cluster of cases [17], are sometimes identified in these low-prevalence Atlantic settings. We have, therefore, studied one of these hotspots in depth in order to produce an epidemiological diagnosis (ED). This ED is a step proposed by the Spanish Wildlife TB Control Plan (PATUBES,[18]), prior to any intervention. ~~It~~ An ED consists in~~implies~~ identifying the species involved and their TB status, along with their distribution, abundance and management within the study area. In this specific case, we have generated information on wildlife and livestock ~~populations~~ numbers and their MTC infection status, carried out interviews with farmers and used participatory Geographic Information System (GIS) [19] to investigate farm-related risk factors for the incidence of TB in cattle. The innovative nature of this study lies in the use of environmental DNA detection as a proxy for exposure risk at the farm (herd) level. Environmental DNA detection is a suitable tool by which to detect contamination with MTC in waterholes and other substrates [20–22]. We hypothesized that the detection of environmental DNA in cattle herds would ~~significantly~~ contribute to risk factor identification at the herd level~~ of herds of cattle and facilitate the ED in our study area~~. ~~We also expected that the presence or absence of MTC in environmental DNA would facilitate the interpretation of TB test results at the herd level.~~

Materials and methods

Study area

The study area is located in the north of the Iberian Peninsula, between Atlantic Spain and the pre-Pyrenean north plateau (centroid coordinates, utm wgs84: 592425, 4743194), to the northwest of Pamplona, in the region of Navarra. This area is a pre-Pyrenean basin that has a marked altitudinal gradient and several rivers and tributaries that form abundant ravines, and is characterized by the presence of the Sierra Urbasa-Andía to the west (a mountainous Natural Park). It occupies an area of approximately 100 Km^2, with an average altitude of 830 meters (range: 384-1260m) and an average

annual temperature of 8º - 12ºC. The livestock management system of the area is largely determined by a marked seasonal variation in temperatures and rainfall. The maximum monthly precipitations are collected in December and the minimum in July. The dominant vegetation comprises Portuguese oak (*Quercus faginea*) and holm oak (*Quercus rotundifolia*) forests, and those of beech (*Fagus sylvatica*) in higher or shady areas. The forests occupy 45% of the area, and cover two thirds of the total surface if we consider scrublands. The remaining third is occupied by pastures, meadows and arable crops.

The land use in the study area is principally determined by the type of property, of which there are three different categories: private land, municipal land and regional land [23]. The private land, which is owned by farmers, is where the barns and warehouses are located. Each municipality has communal land to which every inhabitant of that village has access for livestock grazing and watering. The regional land (Urbasa-Andía Natural Park) has similar rights to those of the municipal land, with the difference that their use extends to all the farmers in the region.

Wildlife densities and TB status

Wild boar densities were assessed using two means. On the one hand, we used three 4x4 photo-trapping grids (16 cameras each, with a distance of 1km between cameras; S1 Fig), installed from September 5 to October 12 (2016), to estimate densities on the basis of an algorithm described previously [24], based on the random detection likelihood of unmarked individuals. We assumed a mean daily speed of 0.115 m/s (own data), a detection radius of 10m and a detection angle of 35º. On the other hand, we used the 2015/16 hunting data to assess the number of wild boar observed per square km. Badger densities were estimated using the same camera-trapping grids and camera specifications and a mean daily speed of 0.021 m/s. This speed was based on data obtained from radio-tracked badgers in Asturias, Spain. Fifty seven hunter-harvested wild boar and one road-killed badger were inspected for visible TB-compatible lesions [25] and submitted for bacteriological culture and molecular

identification in a BSL3 laboratory as described previously [26]. Briefly, approximately 2g of tissue sample was homogenized and decontaminated with an equal volume of 0.75% (w/v) hexadecyl pyridinium chloride solution in agitation during 30 minutes. After centrifugation, pellets were collected with swabs and cultured in Löwenstein-Jensen with sodium pyruvate and Coletsos media (Difco, Spain) at 37ºC up to 3 months. Culture was considered positive when isolates were identified as MTBC by conventional PCR [27] and /or DVR-spoligotyping [28].

Livestock herds, pasture management and TB status

The study area includes 36 cattle herds ~~of cattle~~ with 3,100 cattle in total, 3 herds of goats (a total of 309 goats) and 17 flocks of sheep totaling 4,786 sheep. No pigs are present. With regard to the herds of cattle, 12 were not studied because they were feedlots, because of technical restrictions or owing to their very small size (<10 cattle). The 24 herds selected for the study added 2,786 cattle (89.87% of the total cattle in the study area). All cattle and goats are tested for TB on an annual basis by means of the official single intradermal tuberculin test according to the Spanish Eradication Program of Bovine Tuberculosis [2], and data for the period 2012-2016 was, therefore, kindly provided by the Government of Navarra.

The grazing system is complex, since herds belonging to different owners share pastures and water points. The farms that use the regional pastures (14 of the 24 farms sampled) take their livestock to this land in spring (May) in order to take advantage of these pastures before moving to municipal and private land in summer (August). Those farmers who do not use the regional pastures use the municipal pastures in their place between May and November (9 of the 24 farms sampled). In winter, when it begins to snow, the cattle are moved to stables and fed with fodder or on pastures close to the farm building. Only one farm is limited to using exclusively private land and does not share pastures with other farmers at any time.

All the work performed on the farms was carried out between September 2016 and January 2017, following the recommendations and protocols described in the Spanish Eradication Program of Bovine Tuberculosis [2]. Additionally, the UCLM research ethics committee granted a formal waiver of ethics approval since only routine veterinary care was involved in this study.

On-farm risk assessment

Only two categorical variables (yes/no) were studied, namely the use of regional pastures and the use of municipal pastures. Table 1 lists the quantitative variables considered per cattle farm, and the source of the information.

Table 1. Quantitative variables analyzed, origin of the data, Z statistic and P value (Mann-Whitney's U test) for each of the two criteria defined in this study: Outbreak frequency based on TB testing results and environmental MTC DNA positivity.

		**TB testing** (>1 year positive)		**MTC DNA** (detected)	
**Variable**	**Data source**	**Z**	**P**	**Z**	**P**
Total N of cattle	Official data	1.92	0.056	-1.79	0.078
Number of heifers	Official data	1.80	0.076	-1.30	0.19
N of cattle groups for pasture	Farmer interview	0.64	0.55	0.40	0.69
N of imported cattle (5 years)	Official data	0.02	0.98	-0.73	0.47
N of import movements (5 years)	Official data	0.53	0.59	0.35	0.75
Total land used	Farmer interview	-1.08	0.31	-1.12	0.27
Total own land	Farmer interview	0.82	0.41	0.39	0.72
Total own pastures	Farmer interview	-0.36	0.72	0.71	0.50
Total own woodland	Farmer interview	-0.27	0.82	-0.92	0.42

N own goats	Farmer interview	1.64	0.44	0.24	0.89
N own sheep	Farmer interview	0.65	0.73	-0.51	0.78
N own horses	Farmer interview	1.28	0.20	0.53	0.65
N users’ municipal pastures	Farmer interview	-1.23	0.23	-1.79	0.088
**Farm altitude a.s.l.**	GIS	0.96	0.35	2.42	**0.014**
Farm distance to village	GIS	-1.35	0.18	-1.68	0.10
**Farm distance to woodland**	GIS	1.35	0.18	2.68	**0.006**
N of identified risk points	Farm visit	-0.26	0.81	-1.27	0.21
Freq. sees wild boar	Farmer interview	-0.02	0.98	0	1
Freq. sees roe deer	Farmer interview	-0.24	0.83	-0.56	0.60
Freq. sees badger	Farmer interview	-0.08	0.94	1.34	0.26

Significant results are shown in bold type. a.s.l = at sea level.

Environmental DNA

During January 2017, samples of environmental DNA were taken from cattle from 24 farms. A dry sponge (3M™ Dry-Sponge; 3M-España, Madrid) was pre-hydrated with 15 ml of an isotonic surfactant liquid. Alternatively, 15 ml of sterile distilled water can be used. On each farm, two sponges were scrubbed ten times on the scapula regions of two randomly chosen cattle (one sponge by animal). The scapula region, and no other anatomical region of the animal, was chosen because it is the most accessible area during cattle handling which is relatively close to the brisket, one of the preferred areas for sampling pathogens on cattle hides [29]. In addition, it is a large and quasi-horizontal area, where dust particles can easily deposit. In the laboratory, the sponge was hydrated with 10 ml of the same liquid and centrifuged at 3,500 *g* for 10 minutes. All the steps until recovery of DNA were performed under safety conditions in a BSL3 area.

The pellet was purified as previously described, including mechanical disruption with beads using the fast-prep equipment (before and after the enzymatic lysis) [26]. A real time PCR with which to detect *IS6110* was used to detect MTC in the samples, using the primers (forward 5'-GGTAGCAGACCTCACCTATGTGT-3'; reverse 5'-AGGCGTCGGTGACAAAGG-3') and probe (5' FAM-CACGTAGGCGAACCC-MGB-NFQ 3') described previously [30]. The PCR was performed using the QuantiFast Pathogen +IC Kit, according to the manufacturer's instructions (Qiagen, Hilden, Germany), with a final concentration of 2.5 μM for each primer, 1.25 μM for the probe, and 5 μl of the extracted DNA. All PCR reactions were carried out in a CFX96 TouchTM Real-Time PCR Detection System (Bio-Rad, Hercules, CA, USA) according to the following cycling conditions: 95ºC for 5 minutes followed by 45 2-step cycles at 95ºC for 15 seconds and 60ºC for 30 seconds. Positive (*M. bovis* strain) and negative PCR controls (distilled water) were also included. A conventional PCR was also used to discard the presence of *Mycobacterium avium* subspecies [27]. The analytical specificity of the PCR was tested in 112 DNA samples from pure cultures from 28 *Mycobacterium* species (not belonging to MTC), 5 *Streptomyces sp.*, and one *Lactobacillus brevis* strain. Cross-reaction was detected in few *M. avium subsp. hominissuis* isolates.

Statistics

We decided to use only non-parametric statistics owing to the relatively low sample size (n=24 cattle farms). The Chi square or Fisher's test were used (when appropriate) for categorical data, while Mann-Whitney's U test was employed for numerical data.

Results

The local MTC maintenance host community

The local host community is composed of 3,100 cattle, 309 goats and 4,786 sheep as regards livestock; and between 232 and 431 wild boar (mean density 2.3 to 4.3/km^2 based on photo-trapping and hunting data, respectively) and 91 badgers (photo-trapping; mean density 0.9/km^2), as regards suitable wildlife MTC host species.

Of the 24 cattle farms sampled, eight were negative to the skin test during 2012-2016. The remaining 16 farms had reactors to the skin test with an average of 28.6 positive skin-test animals/year, and none of the reactor farms was consistently positive in all five years (the largest series of consecutive years that a farm was positive was three and four, and this occurred on only two of the 16 reactor farms). This produced a chessboard-like figure of TB incidence in cattle in the study area (S1 Table). All the goats tested during the last five years had obtained negative results to intradermal skin test. However, TB compatible lesions were detected in four sheep as part of the slaughterhouse surveillance, revealing an unknown infection prevalence in this host species.

With regard to wildlife, we sampled 57 hunter-harvested wild boar. The TB-compatible lesion prevalence was 54% (31/57) and the infection prevalence confirmed by culture was 8.8% (5/57). Only one road-killed badger was submitted for culture and was positive. All cattle (N= 7), sheep (N= 4), wild boar (N= 5) and badger (N= 1) isolates obtained in the study were identified as *M. bovis* and were classified with SB0134 spoligotype (according to the Mbovis.org database).

Farm TB status and environmental DNA

Based on the results of the annual intradermal test, the farms were classified as farms with a higher risk, those that were positive to TB for at least two years (consecutive or otherwise) (n=10), and farms with a lower risk, i.e., those with reactors in one year or without reactors (n = 14), in the period of study (2012-2016). Environmental MTC DNA was detected in cattle from 12 of the 24 sampled farms (50% positive farms; 15 positive samples out of 48, 31.3% positive samples). There was no

significant association between the higher risk area and the detection of environmental DNA (Fisher's test, P = 0.982). *Mycobacterium avium* subspecies was only detected in one sample from a farm where the additional sponge was only positive to MTC.

Regional pastures were used by 10 of the 12 MTC DNA positive farms but only by 3/12 MTC DNA negative ones (Fisher's test, P=0.006). The use of municipal pastures was only marginally significant (Fisher's test, P=0.088). With regard to the quantitative variables, altitude and distance to woodland were significant (Mann-Whitney's U test, P=0.014 and P=0.006, respectively. Table 1), indicating that farms at higher altitude~~s~~ and closer to the nearest woodland edge (wildlife habitat) were at higher risk of MTC DNA detection (Fig 2). In contrast, we found no significant difference between "higher risk" and "lower risk" cattle farms as regards using regional pastures or municipal pastures (Fisher's test, P=0.9449 and P=0.704, respectively), and no differences were found for the 20 quantitative variables.

Fig 1. Shared use of communal pastures as a risk factor depending on environmental MTC DNA detection.

Fig 2. Cattle-shelter proximity to woodlands as a risk factor depending on environmental MTC DNA detection. Points represent the different farms studied. Distance expressed in meters.

Discussion

The ~~epidemiological diagnosis~~ED carried out in this tuberculosis hotspot region evidenced that MTC is maintained in the study area owing to a multi-host community composed of domestic and wild hosts (with confirmed infection in ~~at least~~ cattle, sheep, wild boar, and badger~~s~~), despite all the efforts made by the TB eradication program. Environmental sampling has been used as an additional test to

detect mycobacteria on the farms and the results revealed that shared regional pastures and proximity to woodlands were risk factors.

In the study area, about 40 individual cattle become infected annually (based on 2016 official TB testing). Considering that the sensitivity of the single intradermal test is around 70% [31], it would be possible to estimate a higher number of infected cattle (around 52) in this year in the study area. There were, simultaneously, also about 20 to 38 MTC infected wild boar (at a minimum prevalence of 8.8%), plus an unknown number of infected sheep and badgers, sharing the habitat with cattle. It is consequently necessary to assume that MTC, as a paradigmatic multi-host infection, is maintained by a complex matrix of wild and domestic hosts. Any intervention should, therefore, target all possible hosts and not just cattle. Moreover, host populations are not static. The number of wild boar in the study region has, for example, tripled since 1990, and continues to increase [32,33]. Badger abundance is also known to have increased in northern Spain [34]. This and other changes in the composition of the host community, along with the changes in wildlife and livestock management and in livestock numbers, will likely modify the relative importance of the risk factors for shared infections over time.

This study suggests that on-animal environmental DNA sampling can ~~be a proxy for contact (or~~help in assessing contact risk~~)~~ with MTC in livestock, at the herd level. Regional pastures can freely be used by all herds of cattle in this region, while municipal pastures are shared only with farms located in the same municipality. One risk linked to shared pastures is obviously contact with other herds of cattle. However, additional risks as regards regional (or municipal) pastures are the contact with other livestock (in this case, flocks of sheep) and the contact with wildlife. Regional pastures tend to be at higher altitudes and are more suitable for wildlife such as wild boar. In our specific study area, the regional pasture area is, moreover, a protected natural park, where wild boar hunting is restricted. The identification of communal pastures as a risk factor for cattle TB coincides with recent findings obtained from a large-scale survey in Castilla y León, Spain, in which communal pastures were also

found to be a risk factor for wild boar TB [7]. In addition, farm altitude and distance to woodland proved significant (both factors are correlated with each other; Pearson's r = 0.45; P= 0.024)~~02~~. Use of r~~R~~egional pastures is also correlated with distance to woodland, but not with altitude; ANOVA P= 0.021, P= 0.448, respectively). We suggest that ~~closeness to woodland (and in this case, altitude)~~those factors ~~is a~~ are a proxy for the risk of contact with wildlife MTC hosts such as badgers and wild boar. Epidemiological links between badgers, wild boar and cattle have already been evidenced in northern Spain [9,35] and elsewhere [36].

Our results ~~allow~~ prevent us ~~to accept~~from rejecting the initial hypothesis: the detection of environmental DNA in cattle could contribute to risk factor identification at the cattle herd level and, always in combination with the official TB tests of individual animals, could represent a useful epidemiology tool to investigate MTC maintenance in TB hotspot areas. This is of particular relevance given the difficulties involved in interpreting TB testing outcomes in low-prevalence settings [37]. While this finding might, at first glance, appear to be counter-intuitive, it can easily be explained: a positive intradermal official test reflects that cattle have previously come into contact with MTC, and the reactors are almost immediately slaughtered and hence removed from the study herds. In contrast, MTC DNA detection in a herd of cattle, while not necessarily reflecting infection, suggests current contact with an MTC-contaminated source, e.g. wildlife or the environment. ~~We consequently suggest that MTC DNA could be a good proxy for current infection risk.~~ The low prevalence and incidence in the area and the apparently random detection of positive tests could ~~indicate~~ suggest an environmental origin of the new cases. Despite in this study it was not possible to elucidate the true origin of ~~TB~~MTC infection in the studied cattle herds, MTC DNA detection has allowed to ~~point out some specific~~reveal specific risk factors ~~(with an epidemiological sense)~~ on which ~~it would be possible to implement actions in order to avoid new infection~~to act through farm biosafetys ~~We consequently suggest that MTC DNA could be a good proxy for current infection risk.~~

Additional research is needed to verify the usefulness of this ~~novel~~ technique in different situations where its implementation has the potential to support the economic and sanitary viability of livestock farms, such as, for example, in farms with recurrent TB cases, or after stamping out and disinfection of the farm in order to avoid ~~animal~~ reinfection from environmental sources. Additionally, we assume that soil sampling could be a very valuable complementary tool, with which we would have obtained information that would have allowed us to contrast it with our data, thus allowing a more complete view of the epidemiological scenario of the studied region and allowing us to obtain better conclusions. Therefore, additional research is needed, integrating different tools beyond those used in this study that complement the information necessary to carry out comprehensive and useful epidemiological diagnoses.

Additional care is advised for two reasons. First, the real time PCR used to detect MTC DNA is based on the IS*6110* insertion sequence. While all efforts have been made to confirm specificity, such as discarding the presence of *Mycovacterium avium* subspecies, a small risk of cross reaction with some environmental mycobacteria or with other agents, such as certain *Streptomyces* strains, could exist. We assume this limited specificity in some scenarios although this should not invalidate our results. Moreover, *Mycobacterium microti*, a pathogen belonging to the MTC but not included among the main causal agents of bovine TB, will cause positive PCR results [38]. Fortunately, *M. microti* is very rarely reported in Spain (only five records in mycodb.es) [39].

The ~~new~~ tool described herein, which detects MTC DNA at the herd level, opens up new avenues for epidemiological research and facilitates ~~epidemiological diagnosis~~ED. ~~Our aim is to use on-animal environmental DNA as a technique to assess the contact risk of livestock with MTC, not to use it as an alternative to the official diagnostic techniques. In the near future, t~~This tool will hopefully provide additional insights into the herd-level risk factors for contact with MTC and other pathogens in a broad range of settings. ~~We also expect that on-cattle environmental MTC DNA detection will~~

assist in the interpretation of unexpected positive or negative outcomes of the official TB tests in specific epidemiological circumstances.

Acknowledgments

We are indebted to all the farmers who have participated in this study. We are also grateful to all the hunters and field veterinarians who diligently collaborated in sample collection. Last, but by no means least, we would also like to thank the Governments of Navarra's Environmental Service and Forest Guard Section their full collaboration in this project.

References

1. Gortázar C, Che Amat A, O'Brien DJ. Open questions and recent advances in the control of a multi-host infectious disease: Animal tuberculosis. Mamm Rev. 2015;45: 160–175. doi:10.1111/mam.12042

2. Spanish Ministry of Agriculture and Fisheries Food and Environment. Spanish Eradication Program of Bovine Tuberculosis [Internet]. Spain; 2018. Available: https://www.mapa.gob.es/es/ganaderia/temas/sanidad-animal-higiene-ganadera/pnetb_2018_tcm30-436761.pdf

3. Aranaz A, Liébana E, Gómez-Mampaso E, Galán JC, Cousins D, Ortega A, et al. *Mycobacterium tuberculosis* subsp. *caprae* subsp. *nov.*: A taxonomic study of a new member of the Mycobacterium tuberculosis complex isolated from goats in Spain. Int J Syst Bacteriol. 1999;49: 1263–1273. doi:10.1099/00207713-49-3-1263

4. Parra A, Fernández-Llario P, Tato A, Larrasa J, García A, Alonso JM, et al. Epidemiology of *Mycobacterium bovis* infections of pigs and wild boars using a molecular approach. Vet

Microbiol. 2003;97: 123–133. doi:10.1016/j.vetmic.2003.08.007

5. Muñoz-Mendoza M, Romero B, del Cerro A, Gortázar C, García-Marín JF, Menéndez S, et al. Sheep as a Potential Source of Bovine TB: Epidemiology, pathology and evaluation of diagnostic techniques. Transbound Emerg Dis. 2016;63: 635–646. doi:10.1111/tbed.12325

6. Vicente J, Barasona JA, Acevedo P, Ruiz-Fons JF, Boadella M, Diez-Delgado I, et al. Temporal trend of tuberculosis in wild ungulates from mediterranean Spain. Transbound Emerg Dis. 2013;60: 92–103. doi:10.1111/tbed.12167

7. Gortázar C, Fernández-Calle LM, Collazos-Martínez JA, Mínguez-González O, Acevedo P. Animal tuberculosis maintenance at low abundance of suitable wildlife reservoir hosts: A case study in northern Spain. Prev Vet Med. 2017;146: 150–157. doi:10.1016/j.prevetmed.2017.08.009

8. Acevedo P, Romero B, Vicente J, Caracappa S, Galluzzo P, Marineo S, et al. Tuberculosis Epidemiology in Islands: Insularity, Hosts and Trade. PLoS One. 2013;8: e71074. doi:10.1371/journal.pone.0071074

9. Muñoz-Mendoza M, Marreros N, Boadella M, Gortázar C, Menéndez S, de Juan L, et al. Wild boar tuberculosis in Iberian Atlantic Spain: A different picture from Mediterranean habitats. BMC Vet Res. 2013;9: 176. doi:10.1186/1746-6148-9-176

10. Vial F, Johnston WT, Donnelly CA. Local cattle and badger populations affect the risk of confirmed tuberculosis in British cattle herds. PLoS One. 2011;6: e18058. doi:10.1371/journal.pone.0018058

11. Bouchez-Zacria M, Courcoul A, Jabert P, Richomme C, Durand B. Environmental determinants of the *Mycobacterium bovis* concomitant infection in cattle and badgers in

France. Eur J Wildl Res. 2017;63: 74. doi:10.1007/s10344-017-1131-4

12. Acevedo P, González-Quirós P, Prieto JM, Etherington TR, Gortázar C, Balseiro A. Generalizing and transferring spatial models: A case study to predict Eurasian badger abundance in Atlantic Spain. Ecol Modell. 2014;275: 1–8. doi:10.1016/j.ecolmodel.2013.12.011

13. Balseiro A, Rodríguez O, González-Quirós P, Merediz I, Sevilla IA, Davé D, et al. Infection of Eurasian badgers (*Meles meles*) with *Mycobacterium bovis* and *Mycobacterium avium* complex in Spain. Vet J. 2011;190: e21–e25. doi:10.1016/j.tvjl.2011.04.012

14. Acevedo P, Delibes-Mateos M, Escudero MA, Vicente J, Marco J, Gortazar C. Environmental constraints in the colonization sequence of roe deer (*Capreolus capreolus* Linnaeus, 1758) across the Iberian Mountains, Spain. J Biogeogr. 2005;32: 1671–1680. doi:10.1111/j.1365-2699.2005.01310.x

15. Lambert S, Hars J, Réveillaud E, Moyen JL, Gares H, Rambaud T, et al. Host status of wild roe deer in bovine tuberculosis endemic areas. Eur J Wildl Res. 2017;63: 15. doi:10.1007/s10344-016-1071-4

16. Balseiro A, Oleaga Á, Orusa R, Roberto S, Zoppi S, Dondo A, et al. Tuberculosis in roe deer from Spain and Italy. Vet Rec. 2009;164: 468–470. doi:10.1136/vr.164.15.468

17. Lessler J, Azman AS, McKay HS, Moore SM. Perspective piece: What is a hotspot anyway? Am J Trop Med Hyg. 2017;96: 1270–1273. doi:10.4269/ajtmh.16-0427

18. Spanish Ministry of Agriculture and Fisheries F and E. Spanish Wildlife TB Control Plan [Internet]. Spain: Spanish Ministry of Agriculture and Fisheries, Food and Environment; 2017. Available: https://www.mapa.gob.es/es/ganaderia/temas/sanidad-animal-higiene-

ganadera/patubes2017_3_tcm30-378321.pdf

19. Cowie CE, Gortázar C, White PCL, Hutchings MR, Vicente J. Stakeholder opinions on the practicality of management interventions to control bovine tuberculosis. Vet J. 2015;204: 179–185. doi:10.1016/j.tvjl.2015.02.022

20. Barasona JA, Vicente J, Díez-Delgado I, Aznar J, Gortázar C, Torres MJ. Environmental Presence of Mycobacterium tuberculosis Complex in Aggregation Points at the Wildlife/Livestock Interface. Transboundary and Emerging Diseases. 2017: 1148–1158. doi:10.1111/tbed.12480

21. Santos N, Almeida V, Gortázar C, Correia-Neves M. Patterns of *Mycobacterium tuberculosis*-complex excretion and characterization of super-shedders in naturally-infected wild boar and red deer. Vet Res. 2015;46: 129. doi:10.1186/s13567-015-0270-4

22. Barbier E, Boschiroli ML, Gueneau E, Rochelet M, Payne A, de Cruz K, et al. First molecular detection of *Mycobacterium bovis* in environmental samples from a French region with endemic bovine tuberculosis. J Appl Microbiol. 2016;120: 1193–1207. doi:10.1111/jam.13090

23. Cuadrado Iglesias M. Aprovechamientos en común de pastos y leñas. Madrid, ES: Ministerio de Agricultura, Pesca y Alimentación, Servicio de Extensión Agraria; 1980.

24. Rowcliffe JM, Field J, Turvey ST, Carbone C. Estimating animal density using camera traps without the need for individual recognition. J Appl Ecol. 2008;45: 1228–1236. doi:10.1111/j.1365-2664.2008.01473.x

25. Vicente J, Höfle U, Garrido JM, Fernández-De-Mera IG, Juste R, Barral M, et al. Wild boar and red deer display high prevalences of tuberculosis-like lesions in Spain. Vet Res. 2006;37: 107–119. doi:10.1051/vetres:2005044

26. Lorente-Leal V, Liandris E, Castellanos E, Bezos J, Domínguez L, de Juan L, et al. Validation of a real-time PCR for the detection of *Mycobacterium tuberculosis* complex members in Bovine tissue samples. Front Vet Sci. 2019;6: 61. doi:10.3389/fvets.2019.00061

27. Wilton S, Cousins D. Detection and identification of multiple mycobacterial pathogens by DNA amplification in a single tube. Genome Res. 1992;1: 269–273. doi:10.1101/gr.1.4.269

28. Kamerbeek J, Schouls L, Kolk A, Van Agterveld M, Van Soolingen D, Kuijper S, et al. Simultaneous detection and strain differentiation of *Mycobacterium tuberculosis* for diagnosis and epidemiology. J Clin Microbiol. 1997;35: 907–914. doi:10.1016/S0305-4403(02)00239-X

29. Reid CA, Small A, Avery SM, Buncic S. Presence of food-borne pathogens on cattle hides. Food Control. 2002;13: 411–415. doi:10.1016/S0956-7135(01)00050-0

30. Michelet L, de Cruz K, Karoui C, Hénault S, Boschiroli ML. *Mycobacterium microti*, un agent tuberculeux méconnu. In: Dufour Barbara, editor. Épidémiol Et Santé Anim. Maisons-Alfort Cedex, France: Journée scientifique AEEMA, 24 mars 2017; 2017. pp. 71, 129–138.

31. Bezos J, Casal C, Romero B, Schroeder B, Hardegger R, Raeber AJ, et al. Current ante-mortem techniques for diagnosis of bovine tuberculosis. Res Vet Sci. 2014;97: S44–S52. doi:10.1016/j.rvsc.2014.04.002

32. Acevedo P, Quirós-Fernández F, Casal J, Vicente J. Spatial distribution of wild boar population abundance: Basic information for spatial epidemiology and wildlife management. Ecol Indic. 2014;36: 594–600. doi:10.1016/j.ecolind.2013.09.019

33. Leránoz I, Castién E. Evolución de la población del jabalí (*Sus scrofa* L., 1758) en Navarra (N Península Ibérica). Miscel· lània Zoològica. 1996;19: 133–139.

34. Sobrino R, Acevedo P, Escudero MA, Marco J, Gortázar C. Carnivore population trends in Spanish agrosystems after the reduction in food availability due to rabbit decline by rabbit haemorrhagic disease and improved waste management. Eur J Wildl Res. 2009;55: 161–165. doi:10.1007/s10344-008-0230-7

35. Balseiro A, González-Quirós P, Rodríguez Ó, Francisca Copano M, Merediz I, de Juan L, et al. Spatial relationships between Eurasian badgers (*Meles meles*) and cattle infected with *Mycobacterium bovis* in Northern Spain. Vet J. 2013;197: 739–745. doi:10.1016/j.tvjl.2013.03.017

36. Payne A, Chappa S, Hars J, Dufour B, Gilot-Fromont E. Wildlife visits to farm facilities assessed by camera traps in a bovine tuberculosis-infected area in France. Eur J Wildl Res. 2016;62: 33–42. doi:10.1007/s10344-015-0970-0

37. Álvarez J, Pérez A, Marqués S, Bezos J, Grau A, de la Cruz ML, et al. Risk factors associated with negative in-vivo diagnostic results in bovine tuberculosis-infected cattle in Spain. BMC Vet Res. 2014;10: 14. doi:10.1186/1746-6148-10-14

38. Barbier E. Prévalence de *Mycobacterium bovis* dans les agroécosystèmes : analyse de réservoirs environnementaux potentiels (sol, eau douce, faune du sol et faune aquatique) et traçage de la circulation de cette bactérie entre les différents compartiments [Internet]. (Doctoral dissertation). 2016. doi:tel-01476359

39. De Val BP, Sanz A, Soler M, Allepuz A, Michelet L, Boschiroli ML, et al. *Mycobacterium microti* infection in Free-Ranging Wild Boar, Spain, 2017-2019. Emerging Infectious Diseases. Centers for Disease Control and Prevention (CDC); 2019. pp. 2152–2154. doi:10.3201/eid2511.190746

Supporting information

S1 Fig. Location of the photo-trapping grids in the study area.

S1 Table. Official TB testing annual results of the sampled farms. Each column shows the positive animals (cattle) in the respective years, categorized by diagnostic technique according to the following color code: Positive Tuberculin Intradermal Reaction (red); Positive Interferon-γ Gamma Release Assay (blue); Positive Slaughter (Lesions) (yellow); Positive Culture (green). If the same animal has tested positive for more than one diagnostic test, only the color of the most restrictive technique is shown. The last column shows the environmental MTC DNA sampling results categorized as MTC (*Mycobacterium tuberculosis* Complex), MAC (*Mycobacterium avium* Complex), *Mycobacterium* sp. (genus *Mycobacterium* not belonging to MTC or MAC) and 0 (negative or not conclusive).

PONE-D-19-20068R1

Environmental DNA points at shared pastures and proximity to woodlands as tuberculosis risk factors for cattle in multi-host settings

PLOS ONE

Response to Reviewers

Reviewer #1: Thanks for including the suggestions. I believe that paper is now better understood. In any case, as you have clarified, this research is especially a methodological work that must be continued to clarify some issues and prove their true usefulness. Please review references 1 (2015; 45: 160–175.) and 3 (name bacteria in italics).

- ➔ We appreciate the review. Changes made.

Reviewer #2: Dear authors,
Many thanks for responding all my comments included in the first round. I maintain that environmental MTC DNA detection is a promising and an interesting tool to be used in epidemiological studies. However, despite of modification included in this new version, data showed in the manuscript do not support suggestions and conclusions yet, and hence, I cannot recommend publication.
In this work, two criteria have been used to analyse TB status of the farms: TB testing results (based on official data between 2001 and 2016), and on-animal detection of environmental MTC DNA at herd level. Detection of MTC DNA was related with three epidemiological factors (probably correlated among them): altitude of the farm, distance to woodland and the use of regional pasture. However, TB risk level does not correlated with any of the risk factors proposed. In addition, no association was detected between TB risk level (based on data from 2001-2016) and environmental MTC DNA detection at herd level.

- ➔ Thank you very much for offering us a sincere and critical review. It will help us improve the quality of work.

  First, you are correct; there is a correlation between the use of regional pastures and the distance to forest (ANOVA p=0.021), but not with altitude (ANOVA p=0.448). This implies that the different factors that may pose a risk of exposure to mycobacteria are related and, with the data obtained in this study, it is not possible to discern which is the most determining factor.

  However, as they are logically related factors, and with a potential risk of exposure to mycobacteria endowed with a biological sense, we cannot rule out any of them in our epidemiological diagnosis, and we believe that all three should be taken into account to take preventive measures to avoid new infections in cattle herds.

  This new information is added in lines 245-246 and 258-262.

These results are prudently discussed in few sections of the paper. However, authors maintain in the manuscript some statements like "This study suggests that on-animal environmental DNA sampling can be a proxy for contact (or contact risk) with MTC in livestock, at the herd level", "Our results allow us to accept the initial hypothesis: the detection of environmental DNA in cattle could contribute to risk factor identification at the cattle herd level and, always in combination with the official TB tests of individual animals, could represent a useful epidemiology tool to investigate MTC maintenance in TB hotspot areas" or "We consequently suggest that MTC DNA could be a good proxy for current infection risk. The low prevalence and incidence in the area and the apparently random detection of positive tests could indicate an environmental origin of the new cases"; which, from my point of view, are not supported by data showed in the manuscript.

- ➔ Thank you for the comment. In no case, our statements are intended to be overblown and, therefore, have been modified to clarify this point (lines 36-38, 249-250 and 258-262). However, we do believe that the detection of environmental DNA carried out in this study may be a good indicator of the contact risk of cattle with MTC.

To support these suggestions, it would be necessary an association between detection of MTC DNA and TB positivity at herd levels, but it has not been proven. I know that this fact can be due to the sensitivity problems of the diagnostic test used in low prevalence areas. But this fact could be also due to specificity problems of the MTC DNA detection, mainly in areas closed to woodlands where wild boar with a high percentage (26/31 (84%)) of microbiologically unconfirmed TB-like lesions (probably caused by closely related bacteria) are living.
Once validated, on-animal MTC DNA detection would be an useful tool to be implemented in low-prevalence areas like the studied one. However, in order to assess the association between environmental MTC DNA detection and the risk of TB appearance at herd level (based on official diagnosis method), areas with higher prevalence of TB could be better scenarios, allowing to standardize the technique (e.g. establishing percentage or number of animals tested to detect MTC DNA at herd level) and to find out more robust conclusions about the interprtation of this technique.

- ➔ Thank you for the comment. It is true that, a priori, we thought that the detection of environmental DNA could be directly related to the positivity of official tests. However, for biological reasons, the risk of contact with environmental MTC will always be higher than the risk of infection, and we hypothesize that, in areas of low incidence such as the one studied, the detection of environmental DNA from the areas used by livestock would make it easier to establish risk factors than the official tests themselves, which, having such a low and diffuse positivity rate, make it difficult to stablish statistical conclusions.

  In addition, we have also planned to carry out new analyzes based on the official tests subsequent to this study, but, because management actions were implemented to try to reduce the prevalence in the area, the epidemiological scenario changed, and therefore the conclusions could be incorrect.

Reviewer #3: Jordi MARTINEZ-GUIJOSA and collaborators are reporting upon the detection of Mycobacterium tuberculosis complex (MTC) mycobacteria in herds and wild fauna in one region of Spain, where bovine tuberculosis remains of concern.

The topic is of prime interest, yet methods used to address the question are very confusing so that this reviewer had difficulties to draw any firm conclusions from the partial data herein reported.

In details:

1. The tentative integrated approach by the authors (observation of the geography, hygrometry, flora, fauna, etc…) is appropriate and very interesting, but why not sample soil which has been shown to preserve MTC mycobacteria for long, hence a potential source of contamination for the wild and farmed animals? It is a major drawback in the interpretation of reported data; and probably a missed opportunity to better understand the puzzling epidemiology of bovine tuberculosis in that region.

➔ Thank you for the comment. Yes, it is a potential weakness in our study. However, we want to clarify our point of view in this regard.

  Firstly, it should be noted that the objective of this work is to test a novel on-cattle environmental DNA detection methodology. Our hypothesis is that by sampling the environmental DNA deposited on the animals, the environmental sampling is maximized by detecting the mycobacteria present in the cattle herds, which would be a proxy for the mycobacteria present in the places where they have been for the last days or weeks, contributing to risk factor identification at the herd level.

  In addition, when sampling soil (presumably from a point with a potential risk of transmission of the pathogen, such as water or food), it is very difficult to sample it in an integral way (for example, the entire bottom of a water trough or all the banks of a pond), and nor do we make sure to sample the specific risk points that animals use in their normal behavior (it is extensive mountain livestock where animals are poorly managed when they are grazing freely). Through on-cattle environmental DNA detection, we expect that specificity will be lost regarding which specific places are contributing to the transmission of the pathogen, but it allows us to gain sensitivity in terms that we increase the number of relevant points sampled (potentially all recently visited by the herd) at once.

  However, we assume that with soil sampling, we would have obtained additional information that would have allowed us to contrast it with our data, thus allowing a more complete view of the epidemiological scenario of the studied region and allowing us to obtain better conclusions. Therefore, we included this information in the "Discussion" section (lines 269-275) to let the reader know that we consider this weakness in our study.

2. The methods used to assess the presence (absence) of MTC in animals are disparate, not all tested animals have been tested with the same methods and in fact, very few animals have been really tested.

3. Among the methods used to assess the presence of MTC in animals, only culture is appropriate, catching living mycobacteria which are presumably the only ones of interest in the natural history of bovine tuberculosis. In fact, only 57 animals have been tested by culture, and only dead animals have been tested. Why not test feces which are a valuable source for MTC DNA and viable mycobacteria ?

➔ Thank you for the comments 2 and 3. The same method is always used to detect environmental MTC DNA, specifically, this method is described for the first time in this work and represents a new potential tool to help control and eradicate pathogens shared between wildlife and livestock. This methodology is used to infer the risk of contact with environmental MTC DNA at herd level, so it is not intended to establish the presence (absence) of MTC in each animal, and it is not necessary to sample all animals for this purpose.

  Additionally, regarding the methods used in official diagnostic tests at the individual level (each cattle is tested, first by intradermal skin test, in some cases IFN-γ is required, and finally they confirm suspicious tests by culture once slaughtered), we, as scientists, cannot intervene in the official methodologies and protocols, and we can only limit ourselves to use the official data gratefully, which we also consider rigorous and reliable.

  Finally, we did not tested feces because our goal was not to diagnose specific animals, but to try to infer the risk of contact of a herd with mycobacteria through dust deposited on the animals belonging to this herd. This dust comes from places that these cattle have visited naturally throughout their usual pasture behavior.

4. Methods of interest must be detailed for the reader to interpret data:

a. PCR: Negative controls? Internal DNA extraction control ? calibration curve ? Cts values ?

➔ Thank you for the comment. In relation to the controls included in this protocol, positive (*M. bovis* DNA) and negative (distilled water) controls were included for the PCR step. A sentence is included in the manuscript (lines 166): “Positive (*M. bovis* strain) and negative PCR controls (distilled water) were also included.”

  In relation to the calibration curve, this information is not included in the manuscript since an additional manuscript is ready to be sent. To the reviewer´s

knowledge, the PCR was optimized using serial dilutions of a M. bovis DNA. The range of Ct values spanned from an average of 18.83 cycles (S.D. = 0.09) at 6.5 ng/reaction to 39.18 cycles (S.D. = 0.27) at 6.5 fg/reaction. The dynamic range of the reaction spanned from 6.5 ng/reaction to 650 fg/reaction, with a R^2 of 0.999 and an overall efficiency of 100%.

During the extraction step, an additional negative sample (sponge with the surfactant liquid) was included.

b. Culture: culture medium? temperature and atmosphere of incubation ? identification of colonies ?

➔ Thank you for the comment. The references related to the culture protocol have been modified and changed to a recent one where the conditions of decontamination, culture media and confirmation of MTBC by PCR were included. A brief description was also added to the manuscript (lines 110-115): "Briefly, approximately 2g of tissue sample was homogenized and decontaminated with an equal volume of 0.75% (w/v) hexadecyl pyridinium chloride solution in agitation during 30 minutes. After centrifugation, pellets were collected with swabs and cultured in Löwenstein-Jensen with sodium pyruvate and Coletsos media (Difco, Spain) at 37ºC up to 3 months. Culture was considered positive when isolates were identified as MTBC by conventional PCR (Wilton and Cousins, 1992) and /or DVR-spoligotyping ((Kamerbeek et al., 1997)"

https://www.ncbi.nlm.nih.gov/pubmed/1282431

https://www.ncbi.nlm.nih.gov/pmc/articles/PMC229700/

Minor remarks:
1. Epidemiological diagnosis: this new concept is very unclear for the reviewer. In the opinion of the reviewer, the authors made a (limited) diagnosis of bovine tuberculosis, using three different methods. Interpretation of these limited diagnosis data in terms of epidemiology would require a large sample of tested animals, and integration of the sensitivity / specificity values of each one of the three tests, in calculations.

➔ Thank you. Your comment has shown us that we do not make clear the concept of epidemiological diagnosis (ED).
First of all, we want to clarify that we are not only referring to the characterization of the infection in the studied region, but rather we are referring to the study and analysis of the distribution, patterns and risk factors of health and disease conditions in defined populations of the studied region (at the epidemiological level). In this sense, we consider that the novel tool used in this work can help to risk factor identification at the herd level. This point has been clarified in lines70-78: "An ED consists in identifying the species involved and their TB status, along with their distribution, abundance and management within the study area. In this specific case, we have generated information on wildlife and livestock numbers and their MTC infection status, carried out interviews with farmers and used participatory Geographic Information System (GIS) [19]

to investigate farm-related risk factors for the incidence of TB in cattle. The innovative nature of this study lies in the use of environmental DNA detection as a proxy for exposure risk at the farm (herd) level. Environmental DNA detection is a suitable tool by which to detect contamination with MTC in waterholes and other substrates [20–22]. We hypothesized that the detection of environmental DNA in cattle herds would contribute to risk factor identification at the herd level."

2. Line 75: Is detection of "environmental" MTC DNA really innovative?

- ➔ Thank you for the comment. Actually, the detection of environmental MTC DNA is not innovative, what is innovative is the tool used (the pre-hydrated sponges) and the sampling protocol, based on indirect sampling of the environment through sampling the animals that have used the resources in that environment.

3. Line 134: UCLM ?

- ➔ Thank you. Univesidad de Castilla la Mancha (Castilla la Mancha University)
