# Supplementary material for: Environmental DNA: A promising factor for tuberculosis risk assessment in multi-host settings
Source: PLoS One. 2020 May 29;15(5):e0233837. doi: 10.1371/journal.pone.0233837 (PMC7259669; doi:10.1371/journal.pone.0233837)
Supplement: Attachment — Submitted filename: Response to Reviewers.pdf [file pone.0233837.s006.pdf]

PONE-D-19-20068R2

Environmental DNA points at shared pastures and proximity to woodlands as tuberculosis risk factors for cattle in multi-host settings

PLOS ONE

Journal Requirements:

Thank you for submitting your manuscript to PLOS ONE. After careful consideration, we feel that it has merit but does not fully meet PLOS ONE's publication criteria as it currently stands. Therefore, we invite you to submit a revised version of the manuscript that addresses the points raised during the review process.

I am so sorry for the huge delay in sending a decision on your work. The first two referees had a completely differed opinion about the suitability of your work to be accepted for publication. A third referee, however, raised some methodological questions but refused to revise a revised version of your work. To my understanding, you work provided some original and interesting information about the interest of considering environmental DNA in TB epidemiological models. However, your sample size is moderate as well as the contribution of the environmental DNA in your epidemiological model.

- ➔ Thank you for your consideration. We expect that this new version of the manuscript fulfill all expectations.

In consequence, I strongly recommend you to get in touch with a native speaker to help you to put your results into context. I am sure that a good English editing will enhance the impact or your findings.

- ➔ OK, done.

On the other hand, I think that “Environmental DNA a promising factor for tuberculosis risk assessment in multi-host settings” is a more appropriate title for your work.

- ➔ Thank you for the recommendation. The title has changed to “Environmental DNA: a promising factor for tuberculosis risk assessment in multi-host settings”.

Enclosed you will find some additional comments embedded in the pdf.

- ➔ Thank you. All comments have been considered and included into the new version of the manuscript.
