# Supplementary material for: Environmental DNA: A promising factor for tuberculosis risk assessment in multi-host settings
Source: PLoS One. 2020 May 29;15(5):e0233837. doi: 10.1371/journal.pone.0233837 (PMC7259669; doi:10.1371/journal.pone.0233837)
Supplement: Attachment — Submitted filename: Response to the Reviewers_.docx [file pone.0233837.s004.docx]

PONE-D-19-20068R1
**Environmental DNA points at shared pastures and proximity to woodlands as tuberculosis risk factors for cattle in multi-host settings**

PLOS ONE

Response to Reviewers

Reviewer #1: Thanks for including the suggestions. I believe that paper is now better understood. In any case, as you have clarified, this research is especially a methodological work that must be continued to clarify some issues and prove their true usefulness. Please review references 1 (2015; 45: 160–175.) and 3 (name bacteria in italics).

- We appreciate the review. Changes made.

Reviewer #2: Dear authors,
Many thanks for responding all my comments included in the first round. I maintain that environmental MTC DNA detection is a promising and an interesting tool to be used in epidemiological studies. However, despite of modification included in this new version, data showed in the manuscript do not support suggestions and conclusions yet, and hence, I cannot recommend publication.
In this work, two criteria have been used to analyse TB status of the farms: TB testing results (based on official data between 2001 and 2016), and on-animal detection of environmental MTC DNA at herd level. Detection of MTC DNA was related with three epidemiological factors (probably correlated among them): altitude of the farm, distance to woodland and the use of regional pasture. However, TB risk level does not correlated with any of the risk factors proposed. In addition, no association was detected between TB risk level (based on data from 2001-2016) and environmental MTC DNA detection at herd level.

- Thank you very much for offering us a sincere and critical review. It will help us improve the quality of work.

First, you are correct; there is a correlation between the use of regional pastures and the distance to forest (ANOVA p=0.021), but not with altitude (ANOVA p=0.448). This implies that the different factors that may pose a risk of exposure to mycobacteria are related and, with the data obtained in this study, it is not possible to discern which is the most determining factor.

However, as they are logically related factors, and with a potential risk of exposure to mycobacteria endowed with a biological sense, we cannot rule out any of them in our epidemiological diagnosis, and we believe that all three should be taken into account to take preventive measures to avoid new infections in cattle herds.

This new information is added in lines 245-246 and 258-262.

These results are prudently discussed in few sections of the paper. However, authors maintain in the manuscript some statements like “This study suggests that on-animal environmental DNA sampling can be a proxy for contact (or contact risk) with MTC in livestock, at the herd level”, “Our results allow us to accept the initial hypothesis: the detection of environmental DNA in cattle could contribute to risk factor identification at the cattle herd level and, always in combination with the official TB tests of individual animals, could represent a useful epidemiology tool to investigate MTC maintenance in TB hotspot areas” or “We consequently suggest that MTC DNA could be a good proxy for current infection risk. The low prevalence and incidence in the area and the apparently random detection of positive tests could indicate an environmental origin of the new cases”; which, from my point of view, are not supported by data showed in the manuscript.

- Thank you for the comment. In no case, our statements are intended to be overblown and, therefore, have been modified to clarify this point (lines 36-38, 249-250 and 258-262). However, we do believe that the detection of environmental DNA carried out in this study may be a good indicator of the contact risk of cattle with MTC.

To support these suggestions, it would be necessary an association between detection of MTC DNA and TB positivity at herd levels, but it has not been proven. I know that this fact can be due to the sensitivity problems of the diagnostic test used in low prevalence areas. But this fact could be also due to specificity problems of the MTC DNA detection, mainly in areas closed to woodlands where wild boar with a high percentage (26/31 (84%)) of microbiologically unconfirmed TB-like lesions (probably caused by closely related bacteria) are living.
Once validated, on-animal MTC DNA detection would be an useful tool to be implemented in low-prevalence areas like the studied one. However, in order to assess the association between environmental MTC DNA detection and the risk of TB appearance at herd level (based on official diagnosis method), areas with higher prevalence of TB could be better scenarios, allowing to standardize the technique (e.g. establishing percentage or number of animals tested to detect MTC DNA at herd level) and to find out more robust conclusions about the interprtation of this technique.

- Thank you for the comment. It is true that, a priori, we thought that the detection of environmental DNA could be directly related to the positivity of official tests. However, for biological reasons, the risk of contact with environmental MTC will always be higher than the risk of infection, and we hypothesize that, in areas of low incidence such as the one studied, the detection of environmental DNA from the areas used by livestock would make it easier to establish risk factors than the official tests themselves, which, having such a low and diffuse positivity rate, make it difficult to stablish statistical conclusions.

In addition, we have also planned to carry out new analyzes based on the official tests subsequent to this study, but, because management actions were implemented to try to reduce the prevalence in the area, the epidemiological scenario changed, and therefore the conclusions could be incorrect.

Reviewer #3: Jordi MARTINEZ-GUIJOSA and collaborators are reporting upon the detection of Mycobacterium tuberculosis complex (MTC) mycobacteria in herds and wild fauna in one region of Spain, where bovine tuberculosis remains of concern.

The topic is of prime interest, yet methods used to address the question are very confusing so that this reviewer had difficulties to draw any firm conclusions from the partial data herein reported.

In details:

1. The tentative integrated approach by the authors (observation of the geography, hygrometry, flora, fauna, etc…) is appropriate and very interesting, but why not sample soil which has been shown to preserve MTC mycobacteria for long, hence a potential source of contamination for the wild and farmed animals? It is a major drawback in the interpretation of reported data; and probably a missed opportunity to better understand the puzzling epidemiology of bovine tuberculosis in that region.

- Thank you for the comment. Yes, it is a potential weakness in our study. However, we want to clarify our point of view in this regard.

Firstly, it should be noted that the objective of this work is to test a novel on-cattle environmental DNA detection methodology. Our hypothesis is that by sampling the environmental DNA deposited on the animals, the environmental sampling is maximized by detecting the mycobacteria present in the cattle herds, which would be a proxy for the mycobacteria present in the places where they have been for the last days or weeks, contributing to risk factor identification at the herd level.

In addition, when sampling soil (presumably from a point with a potential risk of transmission of the pathogen, such as water or food), it is very difficult to sample it in an integral way (for example, the entire bottom of a water trough or all the banks of a pond), and nor do we make sure to sample the specific risk points that animals use in their normal behavior (it is extensive mountain livestock where animals are poorly managed when they are grazing freely). Through on-cattle environmental DNA detection, we expect that specificity will be lost regarding which specific places are contributing to the transmission of the pathogen, but it allows us to gain sensitivity in terms that we increase the number of relevant points sampled (potentially all recently visited by the herd) at once.

However, we assume that with soil sampling, we would have obtained additional information that would have allowed us to contrast it with our data, thus allowing a more complete view of the epidemiological scenario of the studied region and allowing us to obtain better conclusions. Therefore, we included this information in the “Discussion” section (lines 269-275) to let the reader know that we consider this weakness in our study.

2. The methods used to assess the presence (absence) of MTC in animals are disparate, not all tested animals have been tested with the same methods and in fact, very few animals have been really tested.

3. Among the methods used to assess the presence of MTC in animals, only culture is appropriate, catching living mycobacteria which are presumably the only ones of interest in the natural history of bovine tuberculosis. In fact, only 57 animals have been tested by culture, and only dead animals have been tested. Why not test feces which are a valuable source for MTC DNA and viable mycobacteria ?

- Thank you for the comments 2 and 3. The same method is always used to detect environmental MTC DNA, specifically, this method is described for the first time in this work and represents a new potential tool to help control and eradicate pathogens shared between wildlife and livestock. This methodology is used to infer the risk of contact with environmental MTC DNA at herd level, so it is not intended to establish the presence (absence) of MTC in each animal, and it is not necessary to sample all animals for this purpose.

Additionally, regarding the methods used in official diagnostic tests at the individual level (each cattle is tested, first by intradermal skin test, in some cases IFN-γ is required, and finally they confirm suspicious tests by culture once slaughtered), we, as scientists, cannot intervene in the official methodologies and protocols, and we can only limit ourselves to use the official data gratefully, which we also consider rigorous and reliable.

Finally, we did not tested feces because our goal was not to diagnose specific animals, but to try to infer the risk of contact of a herd with mycobacteria through dust deposited on the animals belonging to this herd. This dust comes from places that these cattle have visited naturally throughout their usual pasture behavior.

4. Methods of interest must be detailed for the reader to interpret data:

a. PCR: Negative controls? Internal DNA extraction control ? calibration curve ? Cts values ?

Thank you for the comment. In relation to the controls included in this protocol, positive (*M. bovis* DNA) and negative (distilled water) controls were included for the PCR step. A sentence is included in the manuscript (lines 166): “Positive (*M. bovis* strain) and negative PCR controls (distilled water) were also included.”

In relation to the calibration curve, this information is not included in the manuscript since an additional manuscript is ready to be sent. To the reviewer´s knowledge, the PCR was optimized using serial dilutions of a M. bovis DNA. The range of Ct values spanned from an average of 18.83 cycles (S.D. = 0.09) at 6.5 ng/reaction to 39.18 cycles (S.D. = 0.27) at 6.5 fg/reaction. The dynamic range of the reaction spanned from 6.5 ng/reaction to 650 fg/reaction, with a R^2^ of 0.999 and an overall efficiency of 100%.

During the extraction step, an additional negative sample (sponge with the surfactant liquid) was included.

b. Culture: culture medium? temperature and atmosphere of incubation ? identification of colonies ?

Thank you for the comment. The references related to the culture protocol have been modified and changed to a recent one where the conditions of decontamination, culture media and confirmation of MTBC by PCR were included. A brief description was also added to the manuscript (lines 110-115): “Briefly, approximately 2g of tissue sample was homogenized and decontaminated with an equal volume of 0.75% (w/v) hexadecyl pyridinium chloride solution in agitation during 30 minutes. After centrifugation, pellets were collected with swabs and cultured in Löwenstein-Jensen with sodium pyruvate and Coletsos media (Difco, Spain) at 37ºC up to 3 months. Culture was considered positive when isolates were identified as MTBC by conventional PCR (Wilton and Cousins, 1992) and /or DVR-spoligotyping ((Kamerbeek et al., 1997)”

<https://www.ncbi.nlm.nih.gov/pubmed/1282431>

<https://www.ncbi.nlm.nih.gov/pmc/articles/PMC229700/>

Minor remarks:
1. Epidemiological diagnosis: this new concept is very unclear for the reviewer. In the opinion of the reviewer, the authors made a (limited) diagnosis of bovine tuberculosis, using three different methods. Interpretation of these limited diagnosis data in terms of epidemiology would require a large sample of tested animals, and integration of the sensitivity / specificity values of each one of the three tests, in calculations.

- Thank you. Your comment has shown us that we do not make clear the concept of epidemiological diagnosis (ED).

First of all, we want to clarify that we are not only referring to the characterization of the infection in the studied region, but rather we are referring to the study and analysis of the distribution, patterns and risk factors of health and disease conditions in defined populations of the studied region (at the epidemiological level). In this sense, we consider that the novel tool used in this work can help to risk factor identification at the herd level. This point has been clarified in lines70-78: “An ED consists in identifying the species involved and their TB status, along with their distribution, abundance and management within the study area. In this specific case, we have generated information on wildlife and livestock numbers and their MTC infection status, carried out interviews with farmers and used participatory Geographic Information System (GIS) [19] to investigate farm-related risk factors for the incidence of TB in cattle. The innovative nature of this study lies in the use of environmental DNA detection as a proxy for exposure risk at the farm (herd) level. Environmental DNA detection is a suitable tool by which to detect contamination with MTC in waterholes and other substrates [20–22]. We hypothesized that the detection of environmental DNA in cattle herds would contribute to risk factor identification at the herd level.”

2. Line 75: Is detection of “environmental” MTC DNA really innovative?

- Thank you for the comment. Actually, the detection of environmental MTC DNA is not innovative, what is innovative is the tool used (the pre-hydrated sponges) and the sampling protocol, based on indirect sampling of the environment through sampling the animals that have used the resources in that environment.

3. Line 134: UCLM ?

- Thank you. Univesidad de Castilla la Mancha (Castilla la Mancha University)
